# Nutritional and immunological evaluation of *Nannochloropsis oculata* as a potential Nile tilapia-aquafeed supplement

**DOI:** 10.1186/s12917-023-03618-z

**Published:** 2023-04-19

**Authors:** Eman Zahran, Samia Elbahnaswy, Fatma Ahmed, Iman Ibrahim, Asmaa A. Khaled, Elsayed A. Eldessouki

**Affiliations:** 1 Department of Aquatic Animal Medicine, Faculty of Veterinary Medicine, Mansoura University, Mansoura, 35516 Egypt; 2 Department of Zoology, Faculty of Science, Sohag University, Sohag, 82524 Egypt; 3 Pathology Department, Faculty of Veterinary Medicine, Mansoura University, Mansoura, 35516 Egypt; 4 Animal and Fish Production Department, Faculty of Agriculture Saba Basha, Alexandria University, Alexandria, Egypt; 5 Department of Fish Health and Diseases, Faculty of Fish Resources, Suez University, Suez, Egypt

**Keywords:** Fish, Fatty acids, Growth, Histomorphology, Immunity, Microalgae

## Abstract

*Nannochloropsis oculata* (*N. oculata*) is a marine microalga containing bioactive compounds and a high omega-3 polyunsaturated fatty acid (ω-3 PUFAs). Therefore, it is very promising for nutraceutical and the functional food industry applications. Three groups of Nile tilapia (forty-five fish/group) were fed on basal diets or diets containing 5% (N5) or 10% (N10) of the microalga *N. oculata* for seven weeks. Fish growth performance, proximate composition, and lipid (fatty acids/ FAs and lipoproteins) profile were estimated. In addition, the expression pattern of some lipid metabolism and immune-relevant genes were assessed. An enhancement in whole body crude protein and growth indices of Nile tilapia was observed on both the supplemented groups N5 and N10. Higher levels of high-density lipoproteins (HDL); and lower levels of the low-density lipoproteins (LDL) were evident in both supplemented groups, while the cholesterol and triglycerides (TG) levels were similar among groups. Ω-3 PUFAs were the significant FAs profile of tilapia fed on *N. oculata*-supplemented diets in terms of eicosapentaenoic acid, docosahexaenoic acid, and n3/n6 ratio. Concerning the gene expression pattern, heat-shock protein70, glutathione-S-transferase, glutathione peroxidase, and interleukin-1β *(IL-1β)* were elevated significantly in both supplemented groups. *IL-10* is only upregulated in the N10 group. The lipid metabolism-related gene expression showed downregulation of only fatty acid synthase (*FAS*) in both supplemented groups, with no statistical changes in Peroxisome proliferator-activated receptor alpha (*PPARα)*. Tumor necrosis factor-*α* (*TNF-α)*, Transforming growth factor-β1 (*TGF-β1),* and the apoptotic related genes [*caspase3* and Proliferating cell nuclear antigen (*PCNA*)] showed insignificant changes among groups. The histopathological examination of the intestine, liver, and spleen supports our findings and confirms the benefits and safeness of *N. oculata* dietary inclusion. Collectively, *N. oculata* is a very promising nutraceutical for improving fish health and sustainability of aquaculture production.

**Graphical Abstract:**

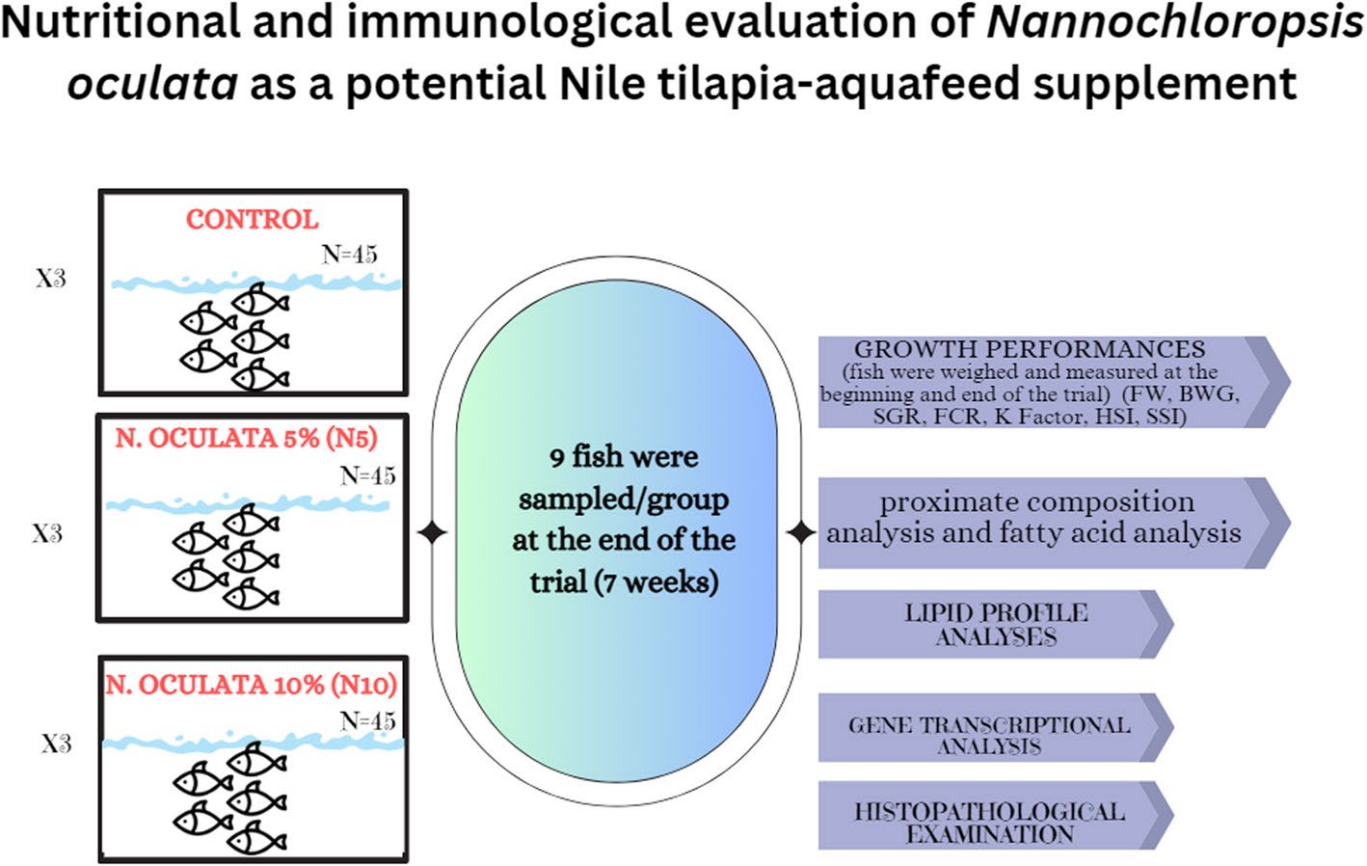

## Introduction

The inclusion of dietary supplements rich in fatty acids (FAs) is an approach of promising benefits for animal health and body composition. However, the imbalance of lipid metabolism by promotion or suppression is the leading cause of lipotoxicity, where the balance between lipolysis and lipogenesis is critical and delicate [1, 2]. Excessive lipolysis and/or lipogenesis via insulin resistance of the adipose tissues were reported to cause lipotoxicity [3]. Subcutaneous fats release plasma free-FAs, mainly during fasting, and the excess fat, triglycerides (TG), accumulate in non-adipose tissues such as the heart, liver, pancreas, and muscle, promoting cell dysfunction since lipids are the primary targets for the reactive oxygen species (ROS), which implies oxidative stress [4, 5]. Therefore, feed supplementation with fat sources should be accurately balanced.

Microalgae are unicellular eukaryotic algae known as resources for FAs, pigments, and other several bioactive metabolites of high nutritive as well as biomedical values [6–8]. They are photosynthetic microorganisms that require low light and nutrients to produce a high amount of energetic biomass and lipids during short periods [9]. They are rich in all the essential amino acids and used as bio-supplies for the polyunsaturated fatty acids (PUFAs), mainly that cannot be synthesized by the vertebrates’ bodies such as C18 PUFA, linoleic (2n-6), and α-linolenic acids (3n-3), which could be then converted into physiologically active long-chain (C20-24) PUFA essential for the normal growth and development [10]. Of them, the marine microalgae *Nannochloropsis* spp., which considered the most promising microalgal feedstock for fish owing to their high rate of biomass accumulation and their high oil, PUFAs, proteins, and carbohydrates content; therefore, they are frequently used in algal nutraceutical, biodiesel, and biofuel production, and the functional food industry applications [11–17]. It contains a high level of FAs, which are affected by nitrogen and phosphorus levels in their growth media, which makes it very promising for nutrition and the applications demanding high lipid concentrations such as biodiesel production and fish dietetics [18–21].

*Nannochloropsis oculata* (*N. oculata*) is characterized by its high productivity, FA, and TG–FA content. In addition,* N*. *oculata* is a strong bio-sourced for the nutraceutical valuable eicosapentaenoic acid and the biodiesel feedstock [22, 23]. The eicosapentaenoic acid (EPA, 20:5n-3) (215 g/kg total fat) and some of the docosahexaenoic acid (DHA, 22:6n-3) (32 g/kg total fat) are the main fatty-acid components of *N. oculata* [24]. Supplementation of fish feed with both FAs improved lipid metabolism, significantly enriched FA profile, and improved fish antioxidant capacity and hematological characteristics [25]. On the other hand, *N. oculata* in fish diets stimulate the production of functional growth hormone [26]. Furthermore, *N. oculata* possessed considerable immunomodulatory efficacy as its sterol rich fraction was found to elicit anti-inflammatory and anti-cancer activities, and its water-soluble polysaccharides showed in vitro immunostimulatory efficacy, which makes it promising for several biomedical applications [27–29]. Further, n3-long chain (LCPUFAs); mainly EPA and DHA could be linked to the membrane fluidity of fish cells [30] that play a role in fish immune defense during inflammatory or other immune responses [31–33]. Thus, the immune response reported in fish can be modified depending on the dietary lipid source [34–36].

To the best of our knowledge, information about the hypolipidemic effect of *N. oculata* is lacking, additionally, our unique approach to fully evaluate the nutritional, biochemical and immunological aspects provide a better understanding for *N. oculata* as an aquafeed supplement.

## Materials and methods

### Ethics statement

All fish in the experimental protocols were reared and handled in accordance with the guidelines of the local Administrative Panel on Laboratory Animal Care and Committee of Mansoura University with the code number (R/108), which specifically approved this study.

#### Experimental fish

One hundred and thirty-five apparently healthy Nile tilapia (*Oreochromis niloticus*) with initial body weight (34.42 ± 1.47 g; mean ± SD) were purchased from a private fish farm in Kafr El-Shiekh governorate, Egypt, and delivered alive to the fish diseases and management laboratory at Mansoura University. Sample size was calculated according to Krejcie and Morgan [37] and a G*Power analysis. In 500 L fiberglass aquariums, fish were acclimated for two weeks. Fish were fed a commercial diet ad libitum at the time (Uccma feed, Egypt; crude protein, 32%; crude lipid, 6.2%; crude fiber, 5.7%). According to Noga [38], evaluations of fish health were conducted during the acclimatization phase, and only fish with a healthy general look and activity level were used. During the period of acclimatization, no clinical symptoms or mortalities were seen.

#### Diet preparation

For the supplemented feed, *N. oculata* dried powder was purchased from the National Research Centre, Cairo, Egypt; the proximate composition and fatty acid profiles of *N. oculata* are presented in Table 1. Three different isonitrogenous and isolipidic diets [non-supplemented, 5% *N. oculata* (N5), or 10% *N. oculata* (N10)] were formulated (Table 2) for the basal and *N. oculata*-supplemented diets. The different diet components were mixed with oil, and water was added to make a stiff dough, extruded through a mincer, and allowed to dry, broken up into pellets, and stored in clean dried plastic bags at 4 °C until use. The fatty acid profiles of experimental diets are presented in Table 3.

**Table 1:** Nutrient composition (crude material %) and fatty acid composition (percentage of total fatty acid methyl esters (FAMEs) of *Nannochloropsis oculata (N. oculata*)

**Nutrient composition of *N. oculata* (%)**		
Crude Protein	46.3	
Crude Lipid	7.5	
Crude ash	11.5	
Crude Fiber	5.5	
Ca	3.25	
P	1.17	
**[% of FAMEs] composition of *Nannochloropsis oculata (N. oculata*)**		
** Saturated fatty acids (SFA)**		
Mystiric acid	C14:0	2.83
Palmitic acid	C16:0	28.81
Stearic acid	C18:0	11.54
** Monounsaturated fatty acids (MUFA)**		
Palmitoleic acid	C16:1n7	3.93
Oleic acid	C18:1n9	10.32
** Polyunsaturated fatty acids (PUFA)**		
** Omega-6**		
Linoleic acid (LA) (ω-6)	C18:2n6	16.93
** Omega-3**		
α-Linolenic acid (ALA) (ω-3)	C18:3n3	10.12
EPA; Eicosapentaenoic acid (ω-3)	C20:5n3	9.16
** ∑n-SFA**		43.18
** ∑n-MUFA**		14.25
** ∑n-6 (ω-6)**		16.93
** ∑n-3 (ω-3)**		19.28
** n3/n6**		1.14

Σ SFA is the sum of saturated fatty acids, Σ MUFA is the sum of monounsaturated fatty acids, Σn-3 is the sum of n-3 polyunsaturated fatty acids, and Σ n-6 is the sum of n-6 polyunsaturated fatty acids

**Table 2:** Ingredients and body composition of basal and experimental isocaloric and isonitrogenous diets (%)

Ingredients (g)	Control	N 5	N 10
**Yellow corn**	19.5	17	16
**Soybean meal**	20	20	17
**Fish meal**	20	17	16
**Corn gluten**	3	3	3
**Gelatin**	2	2	2
**Sunflower oil**	3.5	4	4.16
**Wheat bran**	30.16	30.16	30
**Minerals and vitamins premix**	1	1	1
**Salt**	0.3	0.3	0.3
**Vitamin C**	0.12	0.12	0.12
**Dicalcium phosphate**	0.1	0.1	0.1
**Methionine**	0.32	0.32	0.32
**Algae (*N. oculata*)**	0	5	10
**Proximate analysis (% dry matter basis)**			
** Crude Protein**^**a**^	32.3	31.4	33.2
** Lipid**^**a**^	6.69	6.74	6.86
** Ash**^**a**^	7.75	8.49	7.41
** Ca**^**a**^	1.17	1.17	1.27
** P**^**a**^	0.53	0.53	0.57
** DE (Digestable Energy)**^**b**^ **(kcal/kg)**	3016	3015	3000

The levels of the micro minerals &vitamins for tilapia are covered by supplementation of trace minerals & vitamins premixes as recommended by NRC (2011). Vitamins premix (IU or mg/kg diet); vit. A 5000, Vit.D3 1000, vit. E 20, vit. k3 2, vit. B1 2, vit. B2 5, vit. B6 1.5, vit. B12 0.02, Pantothenic acid 10, Folic acid 1, Biotin 0.15, Niacin 30. Mineral mixture (mg/kg diet); Fe 40, Mn 80, Cu 4, Zn 50, I 0.5, Co 0.2 & Se 0.2

*N. oculataNannochloropsis oculata*

^a^analyzed

^b^calculated value

**Table 3:** Fatty acid composition (percentage of total fatty acid methyl esters (FAMEs) of the experimental diets

**[% of FAMEs]**		**Control**	**N5**	**N10**
**Myristic acid**	MAA	1.84	1.43	2.33
**Palmitic acid**	PA	25.14	20.6	25.16
**Stearic acid**	SA	3.57	3.06	3.47
**Oleic acid**	OA	21.3	18.58	18.48
**Linoleic acid (LA) (ω-6)**	LA	43.52	48.65	42.33
**α-Linolenic acid (ALA) (ω-3)**	α-LA	2.68	3.2	2.58
**EPA; Eicosapentaenoic acid (ω-3)**	EPA	1.15	2.54	3.18
**(DHA); Docosahexaenoic acid (ω-3)**	DHA	0.8	1.9	2.47
**∑SFA**		30.55	25.09	30.96
**∑MUFA**		21.3	18.58	18.48
**∑n-3**		4.63	7.64	8.23
**∑n-6**		43.52	48.65	42.33
**n3/n6**		0.11	0.16	0.19

Σ SFA is the sum of saturated fatty acids, Σ MUFA is the sum of monounsaturated fatty acids, Σn-3 is the sum of n-3 polyunsaturated fatty acids, and Σ n-6 is the sum of n-6 polyunsaturated fatty acids

#### Experimental design

Post 2-weeks acclimation period. Nile tilapia fish were randomly assigned to 3 groups, namely: group 1 (control) fed basal diet, group 2 (N5) fed a diet supplemented with *N. oculata* (5%), and group 3 (N10) fed a diet supplemented with *N. oculata* (10%). Fifteen fish were randomly assigned to each aquarium tank (80 × 40 × 30 cm) (n = 45 fish/group). Electric aerators and an underwater internal power filter were used to maintain the oxygen level in all aquarium tanks. Fish were fed 3% of their biomass (on dry matter basis), repeated every two weeks to readjust the feeding quantity according to National Research Council (NRC) [39] throughout the trial, and 50% of the water was replaced twice a week. Water quality parameters were monitored and maintained throughout the experiment using water quality test kits (Aquarium Pharmaceuticals, Inc.) (temperature 25–27 C, dissolved oxygen 6 mg/L, pH 7.5–8, NH_3_/NH_4_, and nitrite 0.25 mg/L). Daily siphoning of waste material and feces was performed to maintain water quality.

#### Fish growth performance, sampling, and tissue collection

Each fish was weighed and measured at the beginning and end of the trial to determine the growth indices listed below:

$$\mathrm{Weight}\;\mathrm{gain}\;\left(\mathrm g\right)=\mathrm{Mean}\;\mathrm{fina}\;\mathrm{lweight}\;\left(\mathrm g\right)-\mathrm{Mean}\;\mathrm{initial}\;\mathrm{weight}\;\left(\mathrm g\right)$$

$$\mathrm{Specific}\;\mathrm{growth}\;\mathrm{rate}\;(\mathrm{SGR},\%/\mathrm{day})=100\times\lbrack(\mathrm{Ln}\;(\mathrm{mean}\;\mathrm{final}\;\mathrm{body}\;\mathrm{weight})-\mathrm{Ln}\;(\mathrm{mean}\;\mathrm{initial}\;\mathrm{body}\;\mathrm{weight})\rbrack/\mathrm{culture}\;\mathrm{period}\;(\mathrm{days})$$

$$\mathrm{Feed}\;\mathrm{conversion}\;\mathrm{ratio}\;(\mathrm{FCR})=\mathrm{Feed}\;\mathrm{fed}\;(\mathrm g)/\mathrm{Weight}\;\mathrm{gain}\;(\mathrm g)\times100$$

Condition factor according to the following formulae: Condition factor (K) = (W/L3) × 100; where: W = weight of fish in grams and L = total length of fish in "cm".

Nine fish were then collected at random from each group after the trial; six were used for sample collection, and the other three were used for proximate composition analyses. Euthanized fish using buffered MS-222 (Tricaine methanesulfonate, Finquel, Argent) were sampled individually. Blood samples were collected via caudal vein puncture and then transferred to a plain tube to clot at room temperature for 4 h before centrifuging at 1198 × g for 10 min to express serum. Serum samples were stored at -20 °C for subsequent lipid profile analysis. Following that, fish were immediately dissected, liver and spleen were removed and weighed for organosomatic indices calculations using the following formulae: Hepatosomatic indices (HSI) = (Liver w) / W × 100, Splenosomatic indices (SSI) = (Spleen w) / W × 100. The liver was then divided into two portions, one of which was fixed in 10% buffered formalin for histopathological analysis. The other was preserved in RNAlater (Invitrogen, USA) solution and kept at—80 °C until gene transcriptome analysis.

#### Proximate chemical composition analysis of *N. oculata*, feedstuff, and whole fish body

Proximate composition analysis of *N. oculata*, and diets was determined according to the American Association of Cereal Chemists procedure (AACC) [40]; and for the whole fish body was conducted according to the Association of official analytical chemists procedure (AOAC) [41]. The total nitrogen content (N) in the crude protein (N × 6.25) was determined using the Kjeldahl method (1030-Auto-analyzer; Tecator, Tecator, Sweden). The crude lipid concentration was determined following ether extraction using the Soxhlet method (Soxtec System HT6; Tecator). Ash content was determined in the samples using a muffle furnace at 550 °C for 6 h.

#### Fatty acids analyses

Algal extraction and fatty acids analysis was carried out at Central Laboratories Network, National Research Centre, Cairo, Egypt. Briefly, the methylation method described by J Tian, H Ji, H Oku and J Zhou [42], J Folch, M Lees and GH Sloane Stanley [43] was used to extract fatty acids from *N. oculata*, diets, and tissue (whole-body) (based on trichloromethane and methanol). The fatty acid methyl esters (FAMEs) were then determined using an Agilent 7820a Series gas chromatograph (Agilent Technologies) equipped with a gas chromatograph (7890B) and mass spectrometer detector (5977A). The GC was equipped with a DB-WAX column (30 m × 250 μm internal diameter and 0.25 μm film thickness). Analyses were carried out using helium as the carrier gas at a flow rate of 1.9 mL/min at a split ratio of 1:50, injection volume of 1 µL, and the following temperature program: 50 °C for 1 min; rising at 25 °C /min to 200 °C and held for 5 min; rising at 3 °C/min to 220 °C and held for 10 min; rising at 5 °C/min to 240 °C and held for 8 min. The injector and detector were held at 250 °C and 290 °C, respectively. Mass spectra were obtained by electron ionization (EI) at 70 eV and using a spectral range of m/z 60–400 and solvent delay of 1 min. Identification of different constituents was determined by comparing the spectrum fragmentation pattern with those stored in Wiley and NIST Mass Spectral Library data.

#### Lipid profile analyses

Lipoprotein profile, including triglycerides (TG), low-density lipoproteins (LDL), high-density lipoproteins (HDL), and cholesterol, was measured using diagnostic Cobas c pack reagents kits (Roche Diagnostics, Indianapolis, IN, USA) according to the manufacturer’s instructions applied on COBAS INTEGRA 400 plus analyzer (Roche Diagnostics GmbH, USA).

#### Gene transcriptional analysis

According to the manufacturer’s instructions, total RNA was isolated from six liver samples of the Nile tilapia at 7 weeks post-feeding using RNAiso reagent (Takara Bio Inc., Japan). A Nanodrop lite spectrophotometer (Thermo Scientific, US) was used to quantify the RNA concentration at OD 260/280 nm. A cDNA was synthesized using SuperScript III First-Strand Synthesis System with Oligo-dT primers (Invitrogen, USA), according to the manufacturer’s instructions. The RT-qPCR reaction was carried out using Step One Plus ™ Real-time PCR machine (Applied Biosystems, USA) to quantify stress (*Hsp70, GPx, GST*), immune-related genes *(IL1-β, TNF-α, TGF-β1, IL-10*), apoptotic (*caspase3, PCNA*), and lipid metabolism-related genes (*FAS*, *PPARα*). *β*-Actin was included as a housekeeping gene. The primer details were previously published [44–46]. Per the manufacturer’s procedures, each reaction was conducted in a volume of 20 μl via Thunderbird SYBR qPCR Mix reagents (Toyobo, Japan). The amplification program was 95 °C for 1 min, followed by 40 cycles of 95 °C for 15 s and 60 °C for 1 min with a final dissociation analysis step. After the cycling protocol, the melting curves were obtained to assess the specificity. The mRNA expression data were standardized to the *β*-Actin using the 2^−ΔΔCT^ method [47].

#### Histopathological examination

The intestine, liver, and spleen were dissected from Nile tilapia, then collected and fixed in 10% neutral buffered formalin for 24 h. The dissected organs were processed, embedded in paraffin wax, and sliced at 5 µm. The slices were stained using hematoxylin and eosin [48]. The stained slides were examined under a light microscope (Olympus CX 31).

#### Statistical analysis

Data were first subjected to normality and homogeneity checks using Kolmogorov–Smirnov and Levene’s tests, respectively. The significance between the variables of groups was analyzed by a one-way analysis of variance (ANOVA) using the GraphPad Prism® statistics package version 8.4.2 (GraphPad Software, Inc., USA). Normalized individual fold change values were anchored to the lowest value recorded in each data set, and then Log2 transformed, as described previously. Differences were considered statistically significant when *P* < 0.05, *p* < 0.01, and *p* < 0.001. All data were expressed as mean ± standard error of the mean (SEM).

## Results

### Growth performance indices

All growth performance parameters are displayed in Table 4. The FW and BWG of Nile tilapia-fed *N. oculata* at 10% were highly increased compared to the N5 and control groups, with no significant between the latter. SGR of the N10 fish group was significantly increased compared to the control group with no statistical changes to the N5 supplemented group. FCR was significantly better in fish-fed *N. oculata* at 10% than in N5 and control groups, without significance between the latter. K factor, HSI, and SSI showed no significance among groups.

**Table 4:** Growth performance of Nile tilapia fed the experimental diets

Parameter	Feeding Group			*P-*Values		
	**Control**	**N5**	**N10**	**N5/Control**	**N10/Control**	**N10/N5**
**IW**	34.91 ± 1.47	34.36 ± 1.41	34 ± 1.46	-	-	-
**FW**	61.45 ± 2.35^bc^	63.64 ± 2.34^b^	72.27 ± 2.39^a^	0.792	0.008**	0.038*
**BWG**	26.54 ± 2.18^bc^	29.28 ± 2.32^b^	38.27 ± 3.03^a^	0.73	0.007**	0.046*
**SGR**	1.15 ± 0.091^b^	1.259 ± 0.09^ab^	1.55 ± 0.13^a^	0.742	0.032*	0.149
**FCR**	2.14 ± 0.26^a^	1.36 ± 0.13^a^	0.76 ± 0.09^b^	0.149	< 0.001***	0.038
**K Factor**	0.37 ± 0.17^a^	0.22 ± 0.048^a^	0.13 ± 0.062^a^	> 0.999	0.144	0.174
**HSI**	2.35 ± 0.26^a^	2.93 ± 0.26^a^	2.09 ± 0.30^a^	0.302	0.789	0.094
**SSI**	0.17 ± 0.027^a^	0.21 ± 0.023^a^	0.18 ± 0.021^a^	0.441	0.935	0.652

*P< 0.05

**P< 0.01

***P< 0.001

Values with a different letter superscript within the same row indicate a significant difference between groups (*P*< 0.05)

### Proximate composition profiles of *N. oculata*, diets, and fish whole body

The effects of dietary *N. oculata* on the whole-body composition are shown in Table 5. Dietary inclusion was a significant factor, particularly in crude protein (CP) of the fish’s whole body, where CP content increased with the increased percent of dietary *N. oculata*. Tilapia fed 5% *N. oculata* supplemented diet showed no significance (*P* = 0.48), but tilapia fed 10% *N. oculata* supplemented diet recorded a statistically increasing value (*P* = 0.019). No statistically significant changes among groups were recorded on the crude lipid (CL) or Ash contents.

**Table 5:** Proximate composition of Nile tilapia (whole fish body) fed the control and experimental diets

	Nile tilapia fed control diet	Nile tilapia fed N5% diet	Nile tilapia fed N10% diet
CP	17.23 ± 0.67a	18.2 ± 0.51ab	20.3 ± 0.47b
CL	12.8 ± 0.15a	11.6 ± 1.08a	12.3 ± 0.34a
Ash	4.8 ± 0.1538a	4.7 ± 0.18a	5.1 ± 0.37a

Values with a different letter superscript within the same row indicate a significant difference between groups (*P*< 0.05)

### Whole-body fatty acids profile

After seven weeks of feeding with *N. oculata*, considerable changes were recorded in fish’ fatty acid composition compared to those fed with basal diets (Table 6). Three SFA were found in the fish’s whole body, Palmitic (PA), Mystiric (MA), and Stearic acids (SA). In both *N. oculata*-supplemented groups, SA (C18:0) increased (*P* < 0.001), along with the decrease (*P* < 0.001) of MA (C14:0) compared to the control group. A similar significant pattern (*P* < 0.001) of both SA and MA was evident in the N10 group compared to the N5. Nevertheless, the total SFAs demonstrated no statistical difference between groups. Regarding the monounsaturated fatty acids (MUFAs), Oleic acid (OA; C18:1n9) was in the same significant pattern as MA (C14:0). The OA level of the N10 group and the total MUFAs adopt similar patterns to the MA and SFAs, respectively. Furthermore, six polyunsaturated fatty acids (PUFA), three (ω-6) and three (ω-3), were detected in fish bodies. A decrease (*P* > 0.05) was recorded in the level of ω-6 fatty acids of the N10 group compared to the other groups. N5 group recorded the highest significant level of both Arachidonic acid (AA; C20:4n6) (*P* < 0.001) and linoleic acid (LA; C18:2n6) (*P* < 0.01) compared to the other groups, and the N10 group, respectively. The ω-3 fatty acids increased (*P* < 0.001) in both *N. oculata*-supplemented groups in terms of EPA (C20:5n3) and DHA (C22:6n3) levels, being the highest level in the N10 group. Whereas the augmentation of the α-Linolenic acid (ALA; C18:3n3) was higher (*P* < 0.01) only in the N10 than in the control group. Overall, the addition of microalgae significantly influenced the total n‐3 PUFAs profiles, mainly in the N10 group and n‐3/n‐6 ratio at both levels of supplementation.

**Table 6:** Fatty acid composition [percentage of total fatty acid methyl esters (FAMEs)] of Nile tilapia fed the experimental diets

Trivial or (systemic) name	Shorthand name	control	N5%	N10%
**Saturated fatty acids (SFA)**				
** Mystiric acid**	C14:0	3.41 ± 0.03^a^	2.01 ± 0.027^b^	1.09 ± 0.06^c^
** Palmitic acid**	C16:0	27.75 ± 0.42^a^	27.43 ± 0.42^a^	28.13 ± 0.19^a^
** Stearic acid**	C18:0	8.39 ± 0.20^a^	10.56 ± 0.20^b^	15.33 ± 0.19^c^
**Monounsaturated fatty acids (MUFA)**				
** Palmitoleic acid**	C16:1n7	3.75 ± 0.33^a^	2.33 ± 0.33^a^	2.63 ± 0.47^a^
** Oleic acid**	C18:1n9	22.97 ± 0.58^a^	13.58 ± 0.19^b^	11.34 ± 0.20^c^
**Polyunsaturated fatty acids (PUFA)**				
**Omega-6**				
** Linoleic acid (LA) (ω-6)**	C18:2n6	25.13 ± 0.42^ab^	27.1 ± 0.42^a^	12.33 ± 0.19^b^
** Dihomo-γ-linolenic acid (dGLA) (ω-6)**	C18:3n6	1.24 ± 0.23^a^	0.99 ± 0.26^a^	0.37 ± 0.18^a^
** Arachidonic acid (ω-6)**	C20:4n6	0.83 ± 0.05^bc^	1.3 ± 0.06^a^	0.84 ± 0.05^b^
**Omega-3**				
** α-Linolenic acid (ALA) (ω-3)**	C18:3n3	2.29 ± 0.32^a^	4.56 ± 0.19^ab^	7.21 ± 0.19^b^
** EPA; Eicosapentaenoic acid (ω-3)**	C20:5n3	1.47 ± 0.04^a^	2.35 ± 0.03^b^	3.45 ± 0.07^c^
** (DHA); Docosahexaenoic acid (ω-3)**	C22:6n3	3.75 ± 0.19^a^	7.35 ± 0.19^b^	14.63 ± 0.20^c^
**∑n-SFA**		13.18 ± 3.72^a^	13.33 ± 3.74^a^	14.85 ± 3.91^a^
**∑n-MUFA**		13.36 ± 4.30^a^	7.96 ± 2.52^a^	6.99 ± 1.96^a^
**∑n-3(ω-3)**		2.51 ± 0.35^a^	4.75 ± 0.73^ab^	8.43 ± 1.64^b^
**∑n-6(ω-6)**		9.07 ± 4.02^a^	9.79 ± 4.33^a^	4.51 ± 1.96^a^
**n3/n6**		0.28 ± 0.01^a^	0.48 ± 0.01^b^	1.87 ± 0.01^c^

Σ SFA is the sum of saturated fatty acids, Σ MUFA is the sum of monounsaturated fatty acids, Σn-3 is the sum of n-3 polyunsaturated fatty acids, and Σ n-6 is the sum of n-6 polyunsaturated fatty acids

*P< 0.05

**P< 0.01

***P< 0.001

Values with a different letter superscript within the same row indicate a significant difference between groups (*P*< 0.05)

### Fish lipid profile

**Fig. 1:**
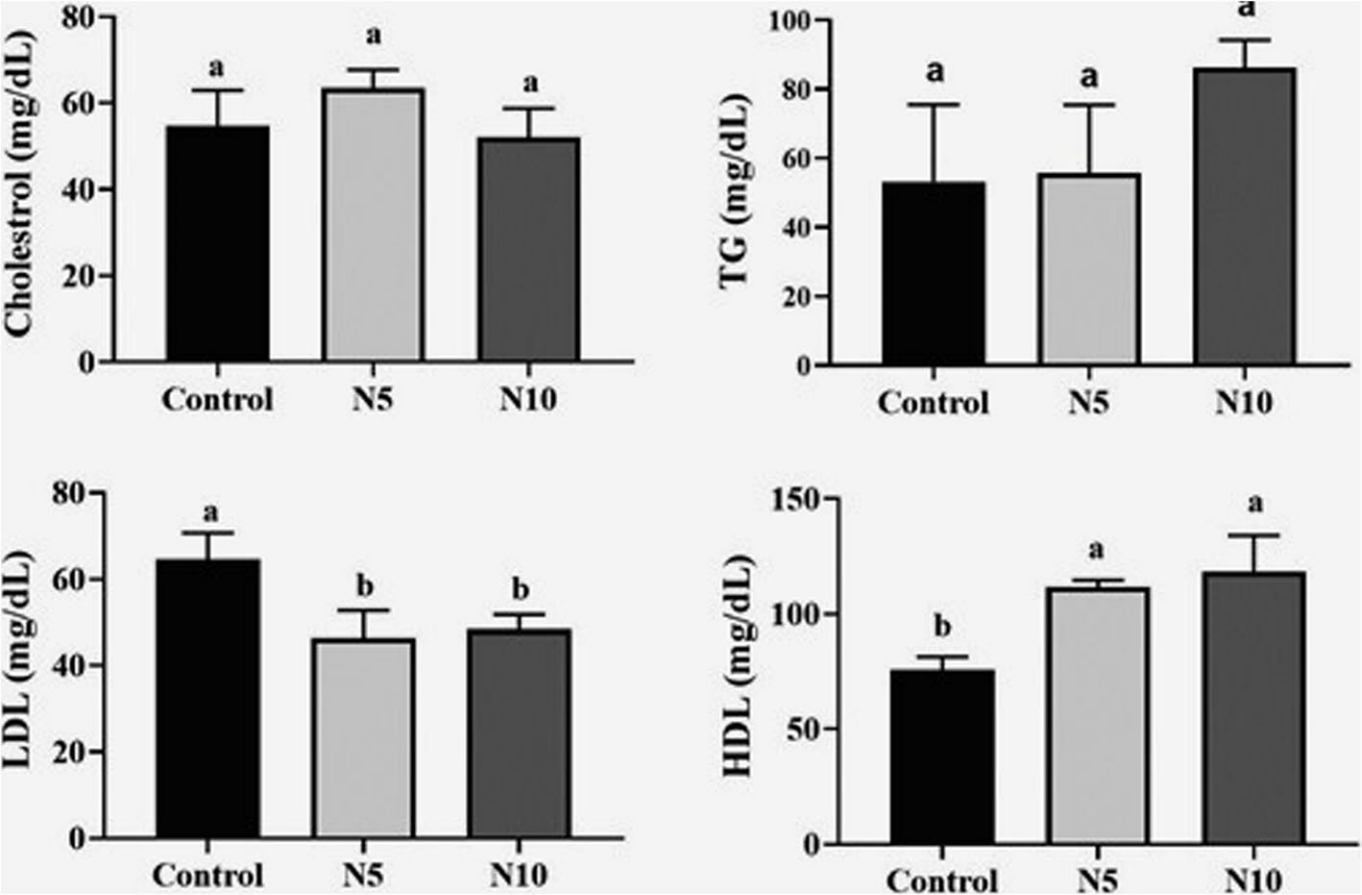
Effects of *Nannochloropsis oculata (N. oculata*) dietary inclusion on serum lipid profile (cholesterol, TG, LDL, and HDL) in Nile tilapia. The fish were fed with the control diet or diets containing *N. oculata* at 5% or 10% for 7 weeks. Data is expressed as the mean ± SEM of six fish. Values with a different letter superscript are significantly different between groups. Significant levels (*P* < 0.05, 0.01, and 0.001), as determined by One-way ANOVA

A noticeable change was observed in the lipid profile of *N. oculata*-supplemented groups, more notably in the N10 group. A significant increase (*P* < 0.01) was observed in the HDL levels, along with a significant decrease (*P* < 0.05) in the LDL levels of both the N5 and N10 groups compared with the control group. No statistical changes were noticed in cholesterol and TG in *N. oculata*-supplemented groups compared to the control one (Fig. 1).

### Effect of *N. oculata* supplementation on hepatic genes expression

**Fig. 2:**
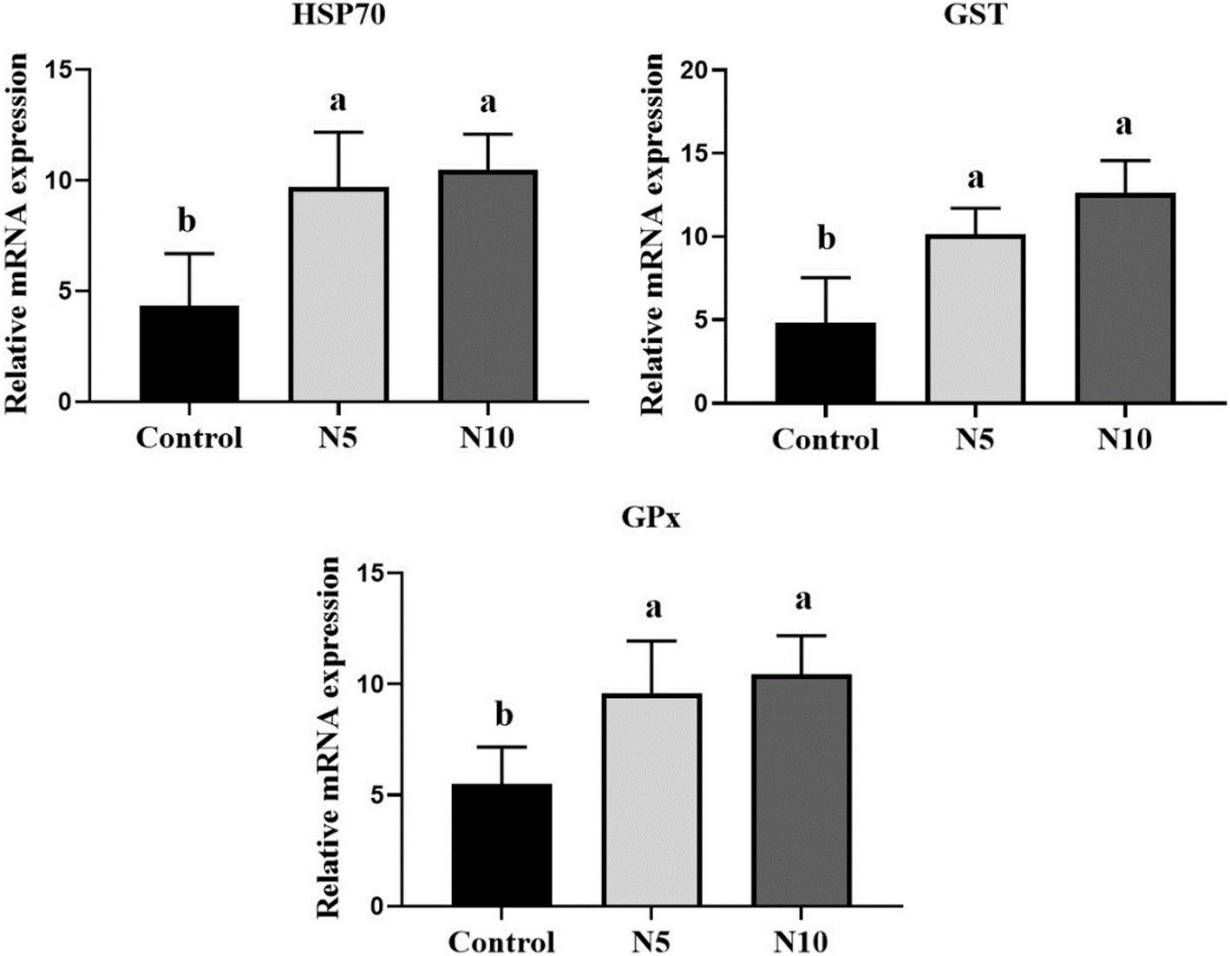
Effects of *Nannochloropsis oculata (N. oculata*) dietary inclusion on the Nile tilapia hepatic gene expression of *HSP70*, *GST*, and *GPx*. The fish were fed with the control diet or diets containing *N. oculata* at 5% or 10% for 7 weeks. Data is expressed as the mean ± SEM of six fish. Values with a different letter superscript are significantly different between groups. Significant levels (*P* < 0.05, 0.01, and 0.001), as determined by One-way ANOVA

**Fig. 3:**
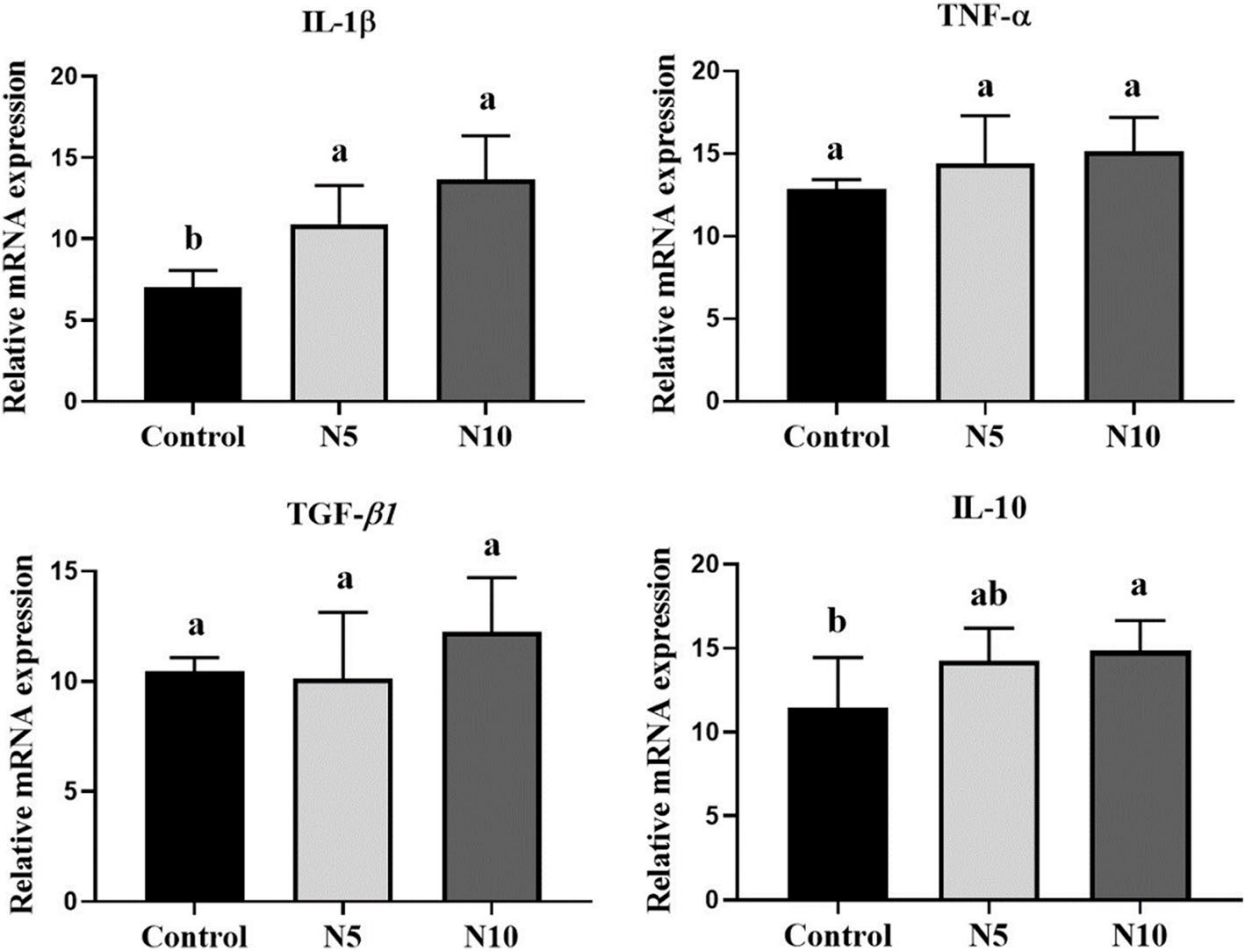
Effects of *Nannochloropsis oculata (N. oculata*) dietary inclusion on the Nile tilapia hepatic gene expression of *IL-1β, TNF-α, TGF-β1, and IL-10*. The fish were fed with the control diet or diets containing *N. oculata* at 5% or 10% for 7 weeks. Data is expressed as the mean ± SEM of six fish. Values with a different letter superscript are significantly different between groups. Significant levels (*P* < 0.05, 0.01, and 0.001), as determined by One-way ANOVA

**Fig. 4:**
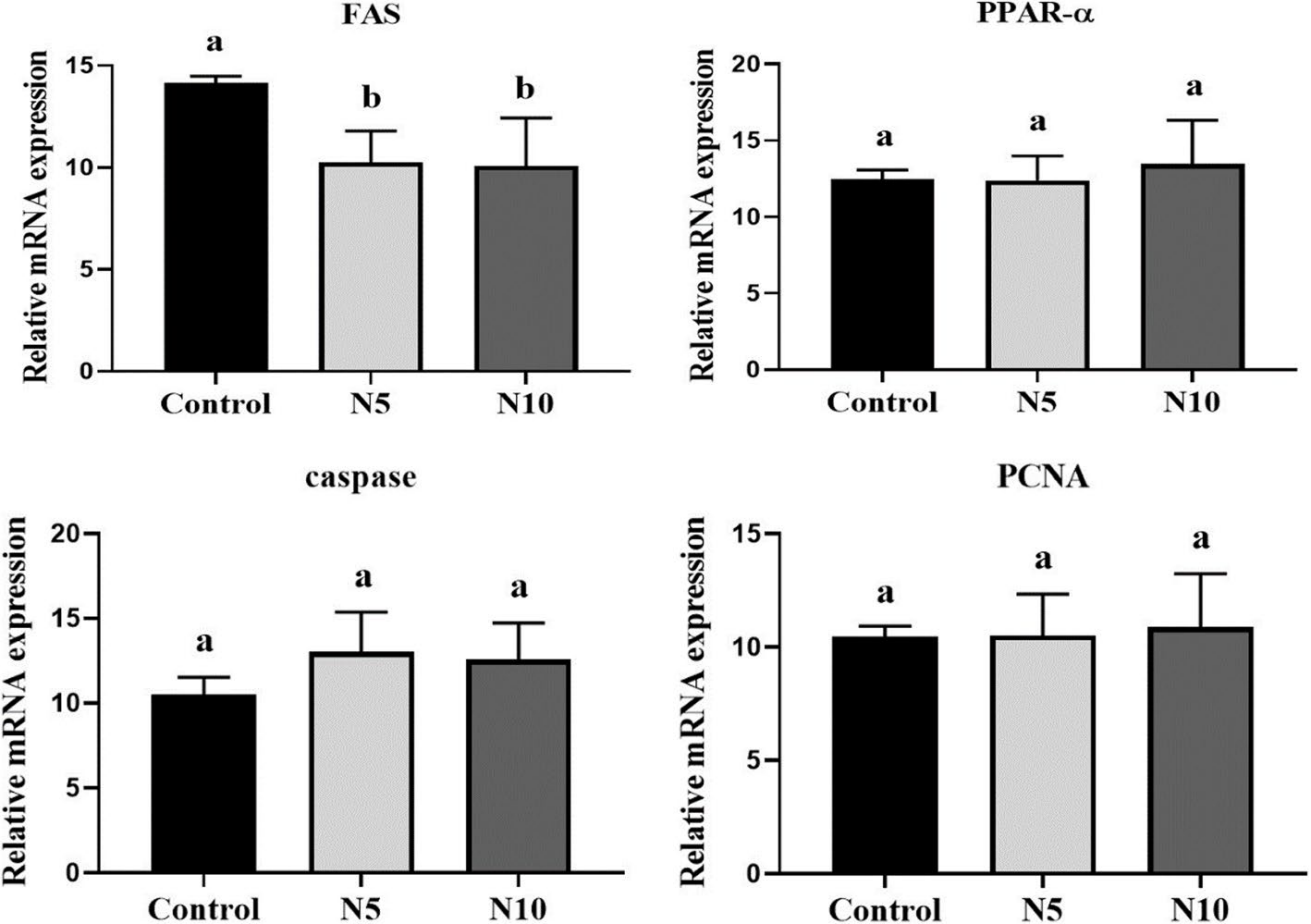
Effects of *Nannochloropsis oculata (N. oculata*) dietary inclusion on the Nile tilapia hepatic gene expression of *FAS, PPARΑ, PCNA, caspase3*. The fish were fed with the control diet or diets containing *N. oculata* at 5% or 10% for 7 weeks. Data is expressed as the mean ± SEM of six fish. Values with a different letter superscript are significantly different between groups. Significant levels (*P* < 0.05, 0.01, and 0.001), as determined by One-way ANOVA

**Fig. 5:**
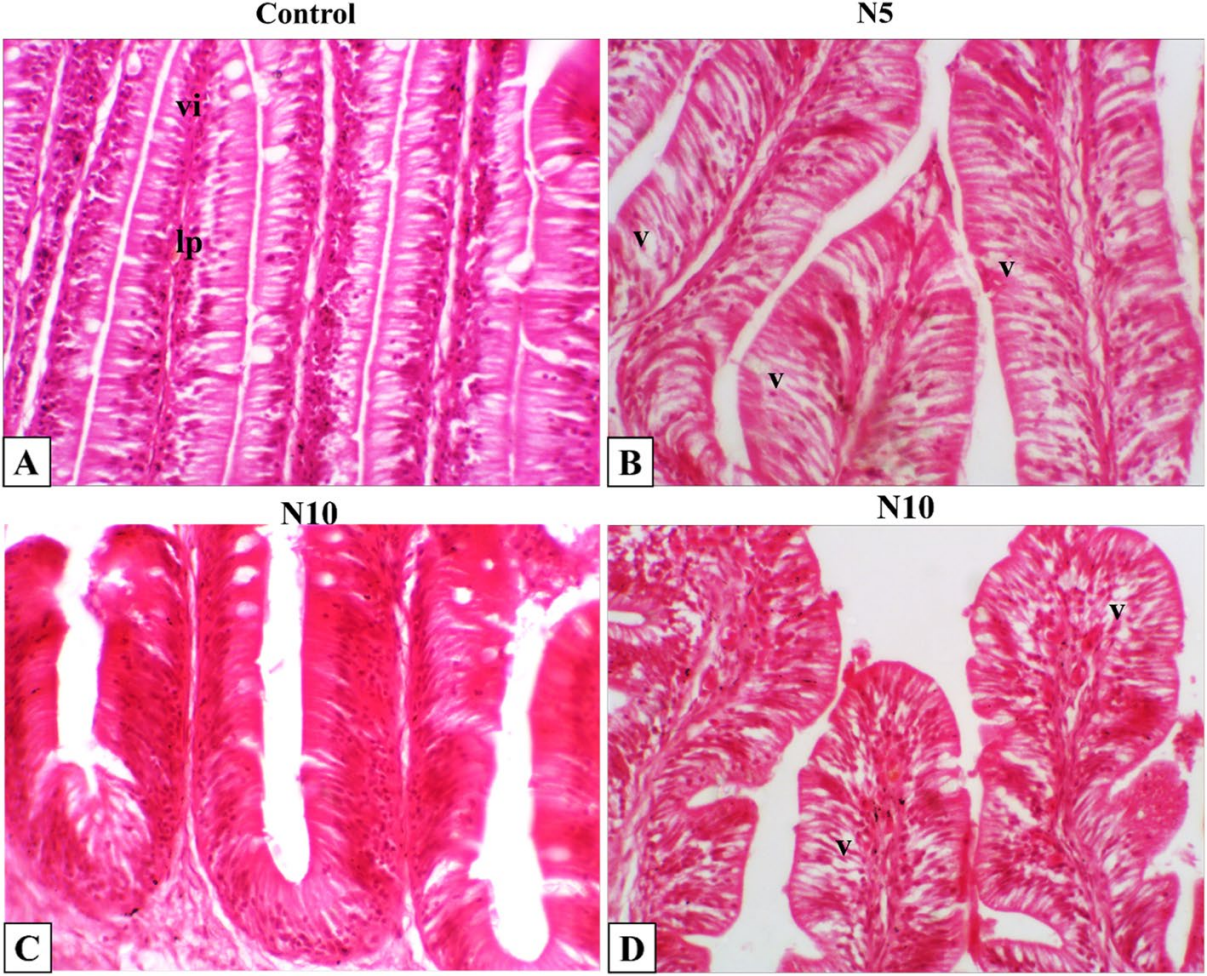
Representative photomicrograph of Nile tilapia intestine. The fish were fed with the control diet or diets containing N. oculata at 5% or 10% for 7 weeks. **A** Control intestine shows normal histological appearance of intestinal villus (vi) and lamina propria (lp). **B** Intestine of tilapia fed on *N. oculata* at 5% showing normal intestinal architecture with minimal intestinal vacuolation (v). Intestine of tilapia fed on *N. oculata* at 10% shows (**C**) Normal histological intestinal mucosa, and (**D**) Other section with minimal intestinal vacuolation (v). H&E, 400X

*N. oculata* supplementation elicited modulated response in the stress and antioxidant-related genes, where the mRNA expression levels of hepatic *HSP70*, *GPx*, and *GST* showed a significant upregulation at both *N. oculata* supplemented levels compared with the control group (*p* < 0.01; *p* < 0.05) (Fig. 2). The expression pattern of the pro-inflammatory (*IL-1β* and *TNF-α*) and anti-inflammatory cytokines (*TGF-β1* and *IL-10*) involved in the immune response is shown in Fig. 3. *IL-1β* was significantly upregulated in both *N. oculata* incorporated groups [N5% (*p* < 0.05); N10% (*p* < 0.01)], however, a nominal increase was noticed for *TNF-α* and *TGF-β1* at both *N. oculata* levels, and at N10%, respectively, compared to control group. On other hand, the *IL-10* expression level was notably upregulated in the N10 group versus the control one (*p* < 0.05). The expression of hepatic genes involved in lipid metabolic pathways in fish-fed *N. oculata* is presented in Fig. 4. The dietary *N. oculata* at 5 and 10% significantly downregulated *FAS* gene expression levels compared to the control group (*p* < 0.01), while insignificant change was noticed in the expression levels of the *PPARα* gene. Concerning the expression of the apoptotic genes, caspase3 and *PCNA*, no significant changes were exhibited among groups (Fig. 5).

### Histopathological findings

**Fig. 6:**
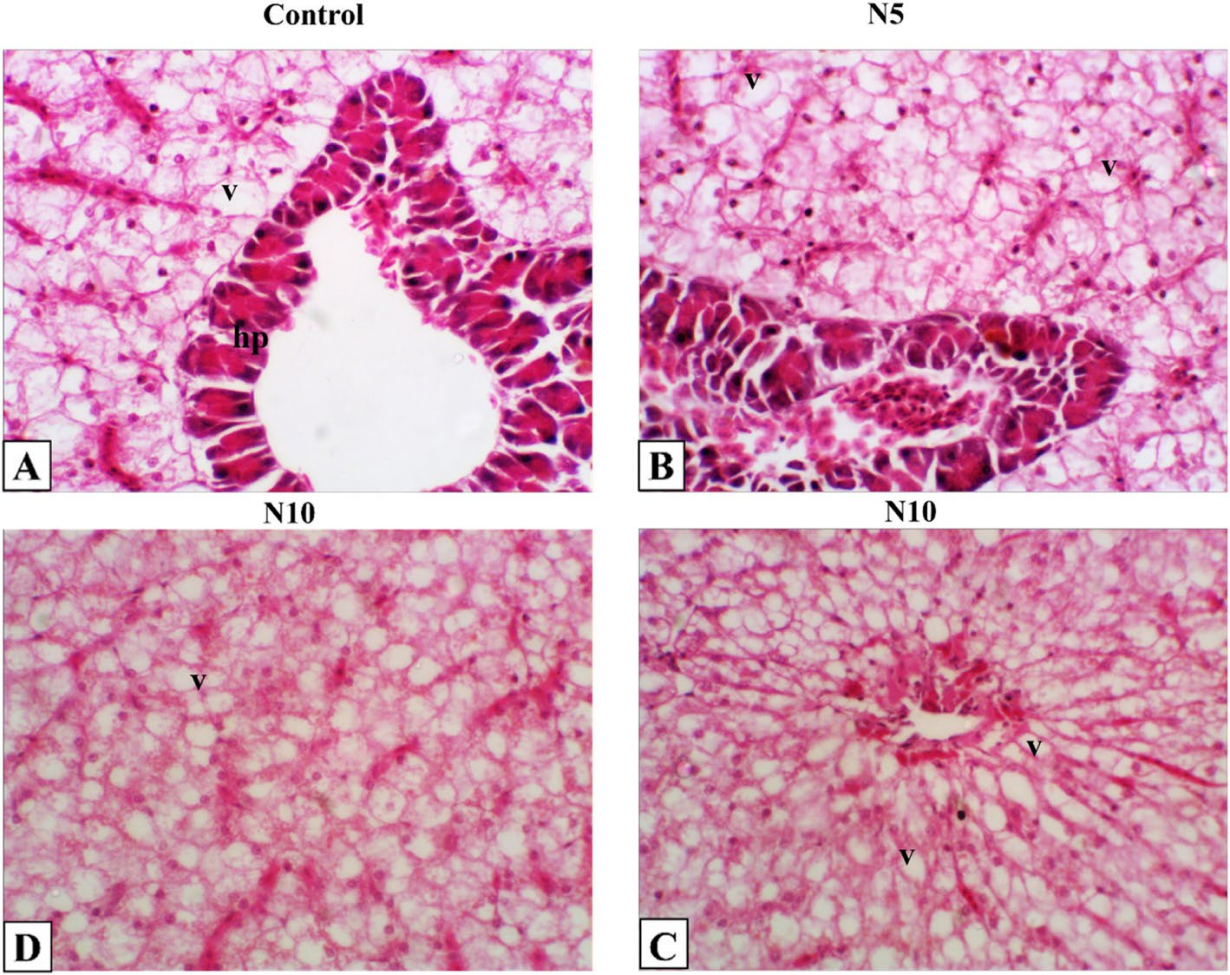
Representative photomicrograph of Nile tilapia liver. The fish were fed with the control diet or diets containing N. oculata at 5% or 10% for 7 weeks. **A** Control liver shows normal histological appearance of hepatopancrease (hp) with vacuolated hepatocyte (v). **B** Liver of tilapia fed on *N. oculata* at 5% shows minimal hepatic vacuolation (v). **C, D** Liver of tilapia fed on *N. oculata* at 10% shows diffuse moderate to severe hepatic vacuolation (v). H&E, 400X

**Fig. 7:**
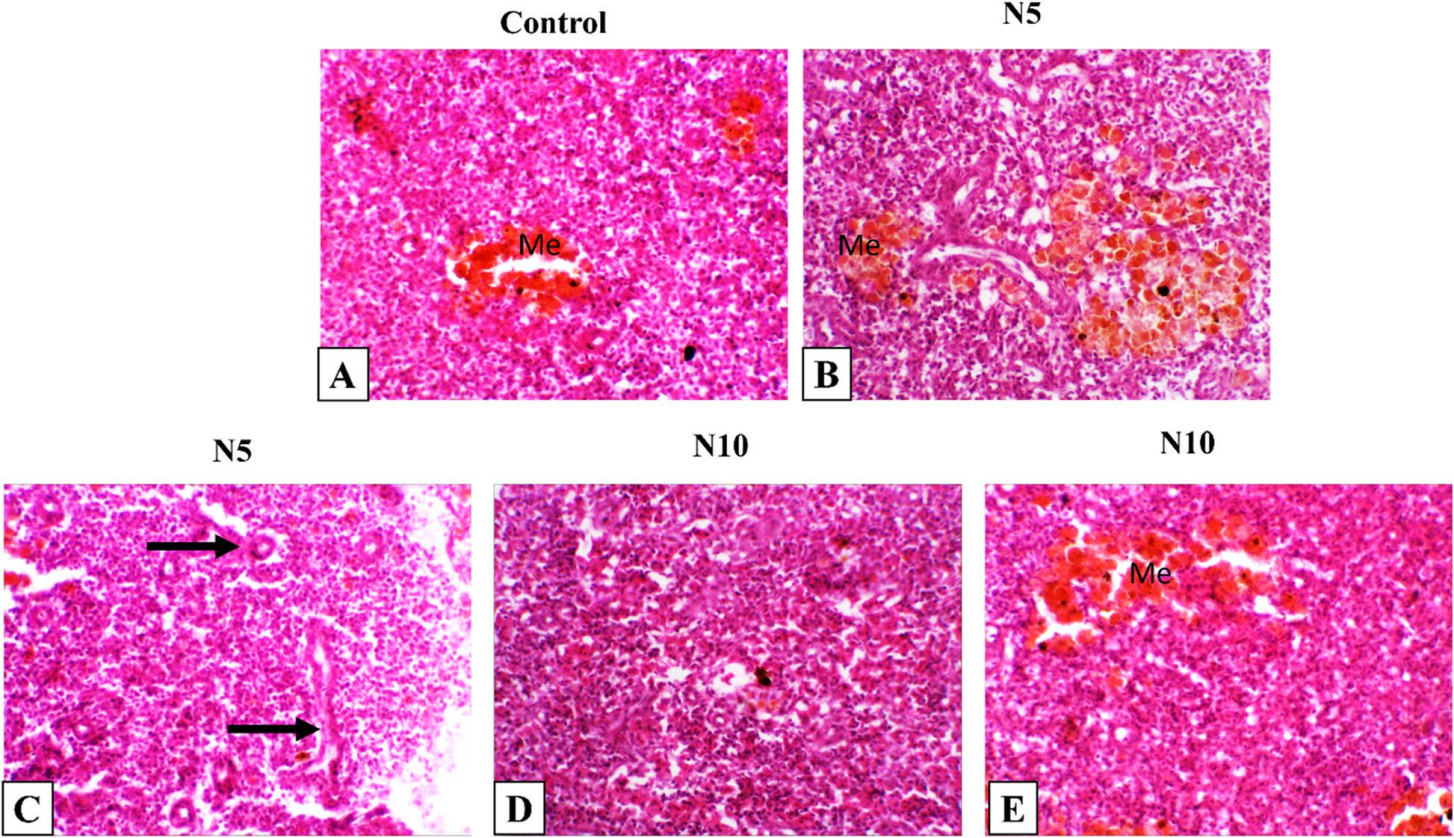
Representative photomicrograph of Nile tilapia spleen. The fish were fed with the control diet or diets containing N. oculata at 5% or 10% for 7 weeks. **A** Control spleen shows mixed red and white pulp and melanomacrophage aggregation, H&E, 400X. Spleen of tilapia fed on *N. oculata* at 5% (**B**) shows extensive, activated melanomacrophage, and (**C**) many reactive endothelial blood vessels (thin arrows). **D, E** Spleen of tilapia fed on *N. oculata* at 10% shows normal histological appearance with minimal lymphoid depletion (thin arrow), other section showing normal architecture with activated melanomacrophages aggregates. H&E, 400X

Histopathological changes in the intestine, liver, and spleen of Nile tilapia were observed in N5, and N10 supplemented groups. The intestine showed typical histological architecture as the control group (Fig. 5A). Intestinal histomorphology was typical, with minimal intestinal epithelial cell vacuolation detected in N5 and N10 groups (Fig. 5B-D). Also, the liver showed normal histological architecture of the hepatopancreas with minimal vacuolated hepatocytes in the control group (Fig. 6A). The same histological appearance of the control liver was seen in N5 and N10 supplemented groups with a slightly increased vacuolation in the N10 group (Fig. 6B-D). The spleen had a normal histological appearance of mixed red and white pulp with central melanomacrophage aggregations (Fig. 7A). Expanded melanomacrophage aggregations and numerous reactive endothelial blood vessels were highly detected in splenic parenchyma of N5 (Fig. 7B, C) than in N10 groups (Fig. 7D, E).

## Discussion

Microalgae were implicated in aquafeed as a good bio-sourced of the essential FAs for the benefit of the aquaculture industry [49–51]. The microalgal-sourced lipids are preferable as the microalgae are low carbon and renewable sources of biomass and chemical feedstock, which provide high bioenergy to the fish being fed [9]. In addition, *O. niloticus* showed high palatability, acceptability, and digestibility of microalgae, including *Nannochloropsis* spp., in its diets [52], which is consistent with our study, where supplementation of Nile tilapia feed with 5% and 10% *N. oculata* enhanced the fish growth performance indices, including FW, BWG, FCR, and SGR. This enhancement, especially in the FCR, indicates the good acceptability and utilization of the *N. oculata* diet by Nile tilapia. In addition, this reflects the adequacy of *N. oculata* amount in Nile tilapia diets, as the excessive algae meal might negatively affect the aquatic animal growth [53].

In an agreement with our findings, Abdelghany et al. [54] reported an enhancement in the Nile tilapia’s growth performance by feeding diets supplemented with 5% *N. oculata* for eight weeks that was better than 15% supplementation. In the same line, 33% replacement of the Nile tilapia meal by *N. oculata* co-product (3%) revealed higher growth performance and body composition [55]. Recently, *N. oculata* replaced soybean at 2.5, 7.5, and 5%; only 2.5% showed the best growth performance [56]. Similarly, dietary inclusion of *Nannochloropsis* sp. (5%) significantly enhanced the antioxidant capacity of the juvenile turbot and (10%) and insignificantly enhanced their weight gain and specific growth rate [57]. On the contrary, this was not the case with Khajepour and Hosseini [58], who reported no improvement in the growth performance of the Atlantic cod supplemented with dietary *Nannochloropsis* spp. up to 30%. Noteworthy, fish species and the morphological structure of its stomach and intestine; the composition of the used *Nannochloropsis* sp., and the basal feed are the main factors affecting the fish’s feed utilization [54]. In addition, the big-sized fish are known for their lower utilization and sensitivity to the nutritional additives than the smaller-sized fish [26].

In the present study, CP was increased in Nile tilapia whole body fed *N. oculata* at 10% compared to other groups. These results reflect the positive influence of the high protein content of *N. oculata* 46.3% supplemented diets. Our result is consistent with previous works that reported growth improvement upon dietary CP-rich microalgae supplementation in juvenile Nile tilapia [55, 59], hybrid red tilapia [60], and channel catfish [61]. The organosomatic indices were similar between fish fed with or without supplemented diets. Stable lipid content of the whole body and HSI suggested no adverse effects of diets on lipid deposition [62]. Since the fatty acid composition of the fish body resembles their feed’s fatty acid composition, fish feed supplementation with a source of essential fatty acids (EFAs) stimulates their growth as EFAs deficiency retards the growth [63]. The current study obtained promising findings of *N. oculata* as an ω-3 PUFA source in the Nile tilapia diets, where the inclusion of *N. oculata* in the Nile tilapia feed was a key feature affecting the ω-3 composition in the fish’s whole body, besides a significant increasing ratio of ω-3/ω-6; this might explain the improvement of fish growth performance, where an augmentation was observed on the fish FW, BWG, and SGR values and on their body content of PUFA.

Our study revealed a marked decrease in LDL levels with a notable increase in HDL levels in the *N. oculata*-supplemented groups and insignificant changes in the TG and cholesterol levels. These results infer that *N. oculata* had a hypolipidemic effect on Nile tilapia via significant contribution of algal EPA and DHA combined with soluble and insoluble fibers as well as phytosterols. The long-chain ω-3 PUFAs were the most predominant in the lipid profile of fish whole-body fed with *N. oculata*. Mainly, microalgae are rich in LCPUFAs [12], which improves their fish body profile level. Noteworthy that the rate of EPA production in the fish body is reflected by the composition of ω-3 PUFA, mainly EPA, in their diets [52]. Elongase and desaturase activities convert EPA to DHA, providing the partial DHA requirements in different species [25], explaining the augmentation of DHA at the expense of EPA levels of N5 and N10 groups. Noteworthy, as DHA is known to decrease the fat [64], its richness in the whole fish body might explain the normal lipid profile and similar HSI [65]. LA and DHA FAs are implicated in methanogenesis suppression and activate nutrient degradability [66], which could explain the enhancement of the fish health and growth performance while keeping their organs’ normal performance. Similar organo-somatic indices (HSI and SSI) and serum lipoproteins (TG and Cholesterol) in *N. oculata*-supplemented groups compared to the control group indicate the normal functional performance of the body organs.

Microalgae contain bio-active micro-minerals, enzyme cofactors implicated in protein synthesis, antioxidation defenses, inflammation amelioration, and multiple physiological functions [66]. In general, diets supplemented with microalgae modulate the fish’s lipid metabolism and stimulate lipase activity [67, 68]. As diets rich in PUFAs decrease liver lipogenesis by suppressing the expression of fatty acid synthase-relevant genes, this is opposed to the diets rich in carbohydrates that stimulate lipogenesis, leading to the elevation of TG levels [1]. To the best of our knowledge, the lipid-lowering effect of *N. oculata* has not been extensively investigated in fish, so we will cite *Nannochloropsis* research studies with other species. *N. oculata* dietary inclusion plays a vital role in the improvement of lipid profile in diabetic rats administered 10 and 20 mg/ kg BW of *N. oculata* for three weeks, where a notable decrease in plasma lipid profile excluding the HDL-C levels was detected; however, in healthy rats, no significant changes in lipid profile were noticed [69]. Microalgae such as *Chlorella vulgaris* (*C. vulgaris*) and *Spirulina platensis* have a lipid-lowering effect, showing an increased HDL and decreased LDL levels upon diet supplementation of African catfish for 12 weeks [70]. In support, a marked decrease in total cholesterol, triglyceride, and LDL levels was revealed in *C. vulgaris*-supplemented Nile tilapia (5%) for one month [71]. Similar effects were observed in common carp-fed *Spirulina* microalgae (3% & 5%) in the diet for 42 days [72].

The liver is one of the main organs involved in fat accumulation, such as PUFAs, which are essential for membrane function in fish, and the liver is generally regarded as the essential tissue for lipogenesis and *β*-oxidation in fish [73]. The expression of hepatic genes related to the antioxidant defense system was analyzed to investigate further the molecular mechanisms of improved antioxidant responses due to *N. oculata*. Results showed that all tested genes encoding antioxidant and immunity (*Hsp70*, *GST*, and *GPx)* responses in the liver were significantly upregulated in Nile tilapia fed with *N. oculata* after 7 weeks. *Nannochloropsis* sp. diminished the lipid peroxidation and enhanced the antioxidant capacity in turbot [57], Atlantic salmon [74], and Nile tilapia [75]. The vital ROS buffering systems, including GPx and GST enzymes, are mainly involved in quenching lipid-peroxidizing chain reactions, reducing H_2_O_2,_ and lipid peroxides as GSH is oxidized to GSSG; this elucidates the ability to protect cells from oxidative damage [76]. Molecular chaperone, *Hsp70*, a biomarker of stress, plays a significant role in the fish’s survival against critical stress response, and thus it is employed as a sensor of cellular redox changes as they have been believed to activate ROS scavengers such as antioxidant enzymes [77]. Besides, it stimulates immune responses [78]. An increase in liver and serum GPx and SOD enzyme activities of juvenile turbot (*Scophthalmus maximus* L.) fed a diet with 5% *N. oceania*-derived defatted meal for 10 weeks, compared to that fish fed on an algae-devoid diet [57]. Besides, in our recent study dietary supplementation of Nile tilapia on *N. oculata* at 5% under naïve conditions for 4 weeks, significantly increased hepatic and intestinal *GST* and intestinal *GPx* genes [75]. In another study, serum SOD and GPx activities recorded the highest values in Nile tilapia fed on a diet partially replaced with *N. oculata* at 5% for 60 days compared to the control fish [56]. Such *GPx* upregulation effect was not evident in the liver of whole *N. oculata*-fed Atlantic Salmon (*Salmo salar*) at 30% for 60 days [79]. Similar induction in the expression of *Hsp70* was evident in the liver of whole *N. oculata* at 10% in Nile tilapia under naïve conditions for 4 weeks [75]. Dietary microalgal *Tribonema* sp. at 5% for 6 weeks notably upregulated mRNA level of *Hsp70* in golden pompano [80]. It has been well documented that natural bioactive compounds in microalgae induced *Hsps* expression in the aquatic organism [81, 82]. Thus, we hypothesized that the potential role of *N. oculata* as an immune response stimulator in Nile tilapia might improve the tolerance of Nile tilapia to various stresses. These results also indicated that the *N. oculata* possessed the highest antioxidant capacities, targeting a prospective new source of natural antioxidants [83]. These results are linked to the increased level of ω-3 PUFAs (α-linolenic, ALA, C18:3 ω3) and eicosapentaenoic (EPA, C20:5 ω3) in the present study, suggesting that ω-3 PUFAs might significantly contribute to *N. oculata* antioxidant activity. Additionally, carotenoids of *N. oculata* have similar antioxidant activity [84]. Thus, the higher gene expression of antioxidant-related genes in the liver of *N. oculata*-fed fish could indicate an improved response to oxidative stress, indicating the importance of microalgal DHA in enhancing the antioxidative enzyme activities to maintain the redox balance [85–87].

Cytokines play an important role in mediating the immune responses interceded by inflammation. Among the cytokines, *IL-1β* and *TNF-α* are potent inflammatory mediators that can fortify secure reactions by mobilizing lymphocytes or by motivating the arrival of a variety of cytokines that initiate macrophages, Natural killers (NK) cells, and lymphocytes to promote the immune response of fish [88]. In the present study, the higher expression of *IL‐1β* (*p* < 0.05, *p* < 0.01) and *TNF-α* (*p* > 0.05) was found in the liver when supplemented with 5 and 10% dietary *N. oculata,* respectively*,* for 7 weeks. Thus, it could be speculated that *TNF-a* was less sensitive than *IL‐1β* to dietary *N. oculata*. Similarly, significant upregulation of *IL‐1β* and *TNF-α* has been demonstrated in Nile tilapia fed the diet with *N. oculata* (5, 10, 15%) for eight weeks [54]. The expression of *IL-1β* and TNF-α genes also increased in Nile tilapia fed with 10% dietary microalgal *Spirulina*, *Arthrospira platensis* for eighty-three days [89]. Furthermore, different fish species and diet formulations could also account for the inconsistent results.

*Nannochloropsis* species are considered a valuable source of n-6 LCPUFAs associated with metabolic pathways related to immune response and inflammation via eicosanoid signaling. Generally, n-6-PUFAs are well known to activate the signaling molecules of nuclear transcription factor-κB (NF-κB), a major transcription factor that promotes pro-inflammatory cytokines genes transcription [90, 91]. Thus, these facts could propose the induction of pro-inflammatory genes in our study after *N. oculata* supplementation, proposing its ability to maintain an active local immune system after the feeding trial. The anti-inflammatory cytokines, *IL-10* and *TGF-β1*, quell the inflammatory response by downregulating the transcriptional levels of cytokine expression [92, 93]. As observed herein, hepatic *IL-10* was upregulated in the N10% group compared to the control group; however, changes were not evident in the hepatic *TGF-β1* gene in both *N. oculata* supplemented groups. A similar expression pattern of hepatic *TGF-β1* has also been reported in Nile tilapia fed the same dose regimen of dietary *N. oculata* under naïve conditions for 4 weeks [75]. These data strengthened the role of *N. oculata* in enhancing the immune response while maintaining its anti-inflammatory prosperities.

To further investigate how *N. oculata* regulated lipid metabolism, the expression of genes related to lipid metabolism in the liver was analyzed. Fatty acid metabolism involves anabolic and catabolic processes catalyzed by key enzymes relevant to its transcriptional factors [94]. Fatty acid synthase (*FAS*) catalyzes the de novo long-chain fatty acid synthesis by converting acetyl-CoA and malonyl-CoA to stearic acid, which is further transformed into monounsaturated fatty acids [95, 96]. Peroxisome proliferator-activated receptor alpha (*PPARα*) has a pivotal role in reducing lipid accumulation by inducing β-oxidation and lipolysis of fatty acids by modulation of gene expression encoding peroxisomal fatty acid-catabolizing enzymes as a transcription factor [97].

No information has been published about evaluating the effects of dietary *N. oculata* levels on mRNA expression of lipid-related genes in fish. *FAS* expression was downregulated in *N. oculata* supplemented groups compared with the control group, while there are no remarkable differences in the *PPARα* expression levels in the same groups. In the same context, hepatic *FAS* gene expression was also diminished in juvenile black seabream fed the higher DHA/EPA ratios (1.60) [25], although the expression of PPAR*-α* was markedly decreased. Similar results were reported in Siberian sturgeon following feeding with high dietary LC n-3HUFA [98]. The mRNA levels of hepatic *FAS* and *PPARα* were not significantly changed in golden pompano fed either microalgal *Tribonema* sp. (1% and 5%) [80] or microalga *Porphyridium* sp. (10% and 50%) for 6 weeks [99].

Dietary PUFAs, especially EPA and DHA, inhibit the transcription of the *FAS* gene and so the hepatic lipogenesis in teleost [73, 100]. Nevertheless, *PPARs* are known to be fatty acid sensors responding to increased cellular fatty acid levels arising from changes in nutritional lipid status [101, 102]. Thus, our data investigated the roles of *N. oculata* supplementation in regulating fatty acid homeostasis via decreasing the *FAS* expression and unchanged *PPARs* genes, eventually leading to less lipid accumulation with subsequent no adverse effect on hepatic β-oxidation of Nile tilapia.

The PCNA, a ring-like protein, provides a regulatory role in DNA replication and cellular metabolism as it is remarkably conserved among eukaryotes [103, 104]. The PCNA has been used as a widespread model in cancer studies and toxicity bioassays in teleost [105, 106]. Besides, caspases are a family of proteins that are one of the main effectors of apoptosis, breaking double-strand DNA [103]. In our study, no marked change in the transcriptional levels of *PCNA* and *caspase*3. After 8 weeks of two different microalgal extracts of 21 and 37% *Phaeodactylum tricornutum*-derived β-glucans, intestinal *PCNA* gene expression was not changed in gilthead seabream juveniles [107]. Results from this study concur with a previous study that demonstrated the transcription level of intestinal *PCNA* was not changed, and lower expression levels of the caspase gene were evident in Atlantic salmon fed a 100% plant oil-based diet, demonstrating that there were no abnormal changes in intestinal cells renewal [103]. Accumulating evidences support that dietary microalgae *Porphyridium* sp. for 6 weeks can diminish caspases mRNA expressions, inhibiting the apoptosis of hepatocytes to protect the liver of juvenile golden pompano [99]. Also, mRNA levels of hepatic *caspase*3, *caspase*6 and *caspase*9 were not significantly affected in golden pompano fed microalgal *Tribonema* sp. (1% & 5%) for 6 weeks [80]. Based on our results, there is no abnormal cell proliferation and apoptotic changes in hepatic tissue of the *N. oculata* groups, suggesting its ability to maintain the hepatic health.

The histological analyses of *N. oculata* dietary supplementation on liver, intestine, and spleen samples did not reveal any considerable negative effect caused by the dietary supplement. The visible vacuoles were seen in all groups. The minor changes in the intestine indicate that *N. oculata* keeps the intestine’s healthy architecture, reflecting its positive effects on the absorption and digestibility capability and thus the nutrient uptake; this also coincided with our finding of the growth performances. The presence of vacuoles in all groups could be attributed to the natural deposition of fat in the liver of Nile tilapia or the high content of fatty acids in *Nannochloropsis* incorporated groups [108]. Furthermore, the spleen shows melanomacrophage activations at 5%and 10% *N. oculata* supplementary levels, indicating immune response enhancement. The absence of inflammatory changes in the liver, intestine, and spleen could be attributed to the significant increase of eicosapentaenoic acid and docosahexaenoic acid (DHA) in *Nannochloropsis* incorporated groups, which play an important role in ameliorating the increase of pro-inflammatory cytokines. Thus, the enhancement of the growth performances, antioxidant activities, and immune response reported in the present study were all histopathologically evident in the intestine, liver, and spleen; and are consistent with previous findings in Nile tilapia and European sea bass [54, 75, 109, 110] emphasizing on the benefits and safety effects of *N. oculata* dietary inclusion on health performance and immunity of Nile tilapia.

Aside from the strengths of our study already mentioned, the CRD design was appropriate for examining the effects of dietary supplements and establishing causality. Additionally, this research helped close a knowledge gap regarding the use of *N. oculata* feed additives as a source of n3-long chain amino acids (LCPUFAs). This study may have some potential limitations, including the inability to generalize the findings to all fish types and the paucity of data on the effects of *N. oculata* feed additives on fish FAs analysis and FAs-related genes. Additional dosing rates of *N. oculata* in feed may also be important to observe a dose-dependent effect and the need to investigate the treatment's effects after a fish pathogen challenge. Therefore, additional research is required to clarify these issues.

## Conclusions

The current study sheds light on the beneficial effects of *N. oculata* dietary inclusion as a protein and lipid source on Nile tilapia growth performance and fatty acid profile. It is a high-quality microalga and a fish oil-saving feed additive. Notably, *N. oculata* incorporation had a positive effect on the expression patterns of antioxidant, cytokine, lipid, and apoptotic-related genes. Thus, aquafeed fortified with *N. oculata* has the potential to improve fish health and increase aquaculture sustainability.

## Data Availability

The data supporting this study’s findings are available from the corresponding author upon reasonable request.

## Abbreviations

*N. oculata*: *Nannochloropsis oculata*
ω-3 PUFAs: Omega-3 polyunsaturated fatty acid
HDL: High-density lipoproteins
LDL: Low-density lipoproteins ()
TG: Triglycerides
*IL-1β*: Interleukin-1β
*FAS*: Fatty acid synthase
*PPAR**α*: Peroxisome proliferator-activated receptor alpha.
*TNF-α*: Tumor necrosis factor-*α*
*TGF-β1*: Transforming growth factor-β1
*PCNA*: Proliferating cell nuclear antigen
FAs: Fatty acids
ROS: Reactive oxygen species
PUFAs: Polyunsaturated fatty acids
EPA: Eicosapentaenoic acid
DHA: Docosahexaenoic acid
NRC: National Research Council
SGR: Specific growth rate
K: Condition factor
HIS: Hepatosomatic indices
SSI: Splenosomatic indices
MS-222: Tricaine methanesulfonate
AACC: American Association of Cereal Chemists
AOAC: Association of official analytical chemists
FAMEs: Fatty acid methyl esters
CP: Crude protein
CL: Crude lipid
EFAs: Essential fatty acids
NK: Natural killers
NF-κB: Nuclear transcription factor-κB

## References

[1] Kersten S (2001). Mechanisms of nutritional and hormonal regulation of lipogenesis. EMBO Rep.

[2] Saponaro C, Gaggini M, Carli F, Gastaldelli A (2015). The subtle balance between lipolysis and lipogenesis: a critical point in metabolic homeostasis. Nutrients.

[3] Burnett V, Jacobs J, Norng S, Ponnampalam E (2016). Feed intake, liveweight gain and carcass traits of lambs offered pelleted annual pasture hay supplemented with flaxseed (*Linum usitatissimum*) flakes or algae (*Schizochytrium* sp.). Anim Prod Sci.

[4] Abele D, Puntarulo S (2004). Formation of reactive species and induction of antioxidant defence systems in polar and temperate marine invertebrates and fish. Comp Biochem Physiol A Mol Integr Physiol.

[5] Morelli M, Gaggini M, Daniele G, Marraccini P, Sicari R, Gastaldelli A (2013). Ectopic fat: the true culprit linking obesity and cardiovascular disease?. s.

[6] Halim R, Webley PA, Martin GJ (2016). The CIDES process: fractionation of concentrated microalgal paste for co-production of biofuel, nutraceuticals, and high-grade protein feed. Algal Res.

[7] Wang H-MD, Li X-C, Lee D-J, Chang J-S (2017). Potential biomedical applications of marine algae. Bioresour Technol.

[8] Bhuvana P, Sangeetha P, Anuradha V, Ali MS (2019). Spectral characterization of bioactive compounds from microalgae: *N. oculata* and *C. vulgaris*. Biocatal Agric Biotechnol.

[9] Beacham TA, Bradley C, White DA, Bond P, Ali ST (2014). Lipid productivity and cell wall ultrastructure of six strains of *Nannochloropsis*: implications for biofuel production and downstream processing. Algal Res.

[10] Jobling M. National Research Council (NRC): Nutrient requirements of fish and shrimp. Washington, DC: The National Academies Press; 2011. ISBN: 978-0-309-16338-5.

[11] Ma Y, Wang Z, Yu C, Yin Y, Zhou G (2014). Evaluation of the potential of 9 *Nannochloropsis* strains for biodiesel production. Bioresour Technol.

[12] Mitra M, Patidar SK, George B, Shah F, Mishra S (2015). A euryhaline *Nannochloropsis gaditana* with potential for nutraceutical (EPA) and biodiesel production. Algal Res.

[13] Cavonius LR, Albers E, Undeland I (2015). pH-shift processing of *Nannochloropsis oculata* microalgal biomass to obtain a protein-enriched food or feed ingredient. Algal Res.

[14] Chua ET, Schenk PM (2017). A biorefinery for *Nannochloropsis*: Induction, harvesting, and extraction of EPA-rich oil and high-value protein. Bioresour Technol.

[15] Liu J, Song Y, Qiu W (2017). Oleaginous microalgae *Nannochloropsis* as a new model for biofuel production: review & analysis. Renewable Sustain Energy Rev.

[16] Samarakoon KW, O-Nam K, Ko J-Y, Lee J-H, Kang M-C, Kim D, Lee JB, Lee J-S, Jeon Y-J (2013). Purification and identification of novel angiotensin-I converting enzyme (ACE) inhibitory peptides from cultured marine microalgae (*Nannochloropsis oculata*) protein hydrolysate. J Appl Phycol.

[17] Zhu Y, Dunford NT (2013). Growth and biomass characteristics of *Picochlorum oklahomensis* and *Nannochloropsis oculata*. J Am Oil Chem Soc.

[18] Rasdi NW, Qin JG (2015). Effect of N: P ratio on growth and chemical composition of *Nannochloropsis oculata* and *Tisochrysis lutea*. J Appl Phycol.

[19] Roy SS, Pal R (2015). Microalgae in aquaculture: a review with special references to nutritional value and fish dietetics. Proceedings of the Zoological Society.

[20] Paes CR, Faria GR, Tinoco NA, Castro DJ, Barbarino E, Lourenço SO (2016). Growth, nutrient uptake and chemical composition of *Chlorella* sp. and *Nannochloropsis oculata* under nitrogen starvation. Lat Am J Aquat Res.

[21] Converti A, Casazza AA, Ortiz EY, Perego P, Del Borghi M (2009). Effect of temperature and nitrogen concentration on the growth and lipid content of *Nannochloropsis oculata* and *Chlorella vulgaris* for biodiesel production. Chem Eng Process Process.

[22] Van Vooren G, Le Grand F, Legrand J, Cuiné S, Peltier G, Pruvost J (2012). Investigation of fatty acids accumulation in *Nannochloropsis oculata* for biodiesel application. Bioresour Technol.

[23] Mitra M, Patidar SK, Mishra S (2015). Integrated process of two stage cultivation of *Nannochloropsis* sp. for nutraceutically valuable eicosapentaenoic acid along with biodiesel. Bioresour Technol.

[24] Hulatt CJ, Wijffels RH, Bolla S, Kiron V (2017). Production of fatty acids and protein by *Nannochloropsis* in flat-plate photobioreactors. PLoS One.

[25] Jin M, Monroig Ó, Lu Y, Yuan Y, Li Y, Ding L, Tocher DR, Zhou Q (2017). Dietary DHA/EPA ratio affected tissue fatty acid profiles, antioxidant capacity, hematological characteristics and expression of lipid-related genes but not growth in juvenile black seabream (*Acanthopagrus schlegelii*). PLoS One.

[26] Chen HL, Li SS, Huang R, Tsai HJ (2008). Conditional production of a functional fish growth hormone in the transgenic line of *Nannochloropsis oculata* (*Eustigmatophyceae*). J Phycol.

[27] Yanuhar U (2015). Effects of Pigment-Protein Fraction from *Nannocloropsis Oculata* on TNFα and IL-6 which Act as an Anti-Inflammatory Against Viral Nervous Necrosis (VNN) Infection. Proc Chem.

[28] Sanjeewa KKA, Fernando IPS, Samarakoon KW, Lakmal HHC, Kim E-A, Kwon O-N, Dilshara MG, Lee J-B, Jeon Y-J (2016). Anti-inflammatory and anti-cancer activities of sterol rich fraction of cultured marine microalga *Nannochloropsis oculata*. Algae.

[29] Pandeirada CO, Maricato E, Ferreira SS, Correia VG, Pinheiro BA, Evtuguin DV, Palma AS, Correia A, Vilanova M, Coimbra MA (2019). Structural analysis and potential immunostimulatory activity of *Nannochloropsis oculata* polysaccharides. Carbohydr Polym.

[30] Snyder RJ, Hennessey TM (2003). Cold tolerance and homeoviscous adaptation in freshwater alewives (*Alosa pseudoharengus*). Fish Physiol Biochem.

[31] Wall R, Ross RP, Fitzgerald GF, Stanton C (2010). Fatty acids from fish: the anti-inflammatory potential of long-chain omega-3 fatty acids. J Nutr Rev.

[32] Calder PC (2017). Omega-3 fatty acids and inflammatory processes: from molecules to man. J Biochem Soc Trans.

[33] Buoite Stella A, Gortan Cappellari G, Barazzoni R, Zanetti M (2018). Update on the impact of omega 3 fatty acids on inflammation, insulin resistance and sarcopenia: A review. J Int J Mol Sci.

[34] Mesa‐Rodriguez A, Hernández‐Cruz CM, Betancor MB, Fernández‐Palacios H, Izquierdo MS, Roo J (2018). Effect of increasing docosahexaenoic acid content in weaning diets on survival, growth and skeletal anomalies of longfin yellowtail (*Seriola rivoliana*, Valenciennes 1833). J Aquac Res.

[35] Zhu L-y, Nie L, Zhu G, Xiang LX, Shao JZ (2013). Advances in research of fish immune-relevant genes: a comparative overview of innate and adaptive immunity in teleosts. J Dev Comp Immunol.

[36] Nguyen TM, Mandiki SN, Gense C, Tran TNT, Nguyen TH, Kestemont P (2020). A combined in vivo and in vitro approach to evaluate the influence of linseed oil or sesame oil and their combination on innate immune competence and eicosanoid metabolism processes in common carp (*Cyprinus carpio*). Dev Comp Immunol.

[37] Krejcie RV, Morgan DW (1970). Determining sample size for research activities. J Educ Psychol Measur.

[38] Noga EJ. Fish disease: diagnosis and treatment, 2nd edition. Hoboken, New Jersey: Wiley-Blackwell; 2010.

[39] National Research Council (NRC). Nutrient Requirements of Fish and Shrimp. Washington, DC: The National Academies Press; 2011. ISBN 978-0-309-16338-5.

[40] AACC. Approved Method of the AACC, 10th ed. American Association of Cereal Chemists, editor. St. Paul: American Association of Cereal Chemists; 2000.

[41] AOAC (2000). Association of official analytical chemists. Official methods of analysis.

[42] Tian J, Ji H, Oku H, Zhou J (2014). Effects of dietary arachidonic acid (ARA) on lipid metabolism and health status of juvenile grass carp, *Ctenopharyngodon idellus*. J Aquac.

[43] Folch J, Lees M, Sloane Stanley GH (1957). A simple method for the isolation and purification of total lipids from animal tissues. J J Biol Chem.

[44] Zahran E, Elbahnaswy S, Risha E, El-Matbouli M (2020). Antioxidative and immunoprotective potential of *Chlorella vulgaris* dietary supplementation against chlorpyrifos-induced toxicity in Nile tilapia. Fish Physiol Biochem.

[45] Standen B, Peggs D, Rawling M, Foey A, Davies S, Santos G, Merrifield D (2016). Dietary administration of a commercial mixed-species probiotic improves growth performance and modulates the intestinal immunity of tilapia, *Oreochromis niloticus*. J Fish Shellfish Immunol.

[46] Alkaladi A (2019). Vitamins E and C ameliorate the oxidative stresses induced by zinc oxide nanoparticles on liver and gills of *Oreochromis niloticus*. Saudi J Biol Sci.

[47] Livak KJ, Schmittgen TD (2001). Analysis of relative gene expression data using real-time quantitative PCR and the 2(-Delta Delta C(T)) Method. Methods.

[48] Roberts RJ. Fish pathology, 4th ed. Oxford: Wiley-Blackwell; 2012.

[49] Miller MR, Nichols PD, Carter CG (2007). Replacement of fish oil with thraustochytrid *Schizochytrium* sp. L oil in Atlantic salmon parr (*Salmo salar* L) diets. Comp Biochem Physiol A Mol Integr Physiol.

[50] Shah MR, Lutzu GA, Alam A, Sarker P, Chowdhury MK, Parsaeimehr A, Liang Y, Daroch M (2018). Microalgae in aquafeeds for a sustainable aquaculture industry. J J Appl Phycol.

[51] Lu Q, Yang L, Deng X (2020). Critical thoughts on the application of microalgae in aquaculture industry. Aquaculture.

[52] Gbadamosi OK, Lupatsch I (2018). Effects of dietary *Nannochloropsis salina* on the nutritional performance and fatty acid profile of Nile tilapia, *Oreochromis niloticus*. Algal Res.

[53] Xiao F, Xing J, Li H, Xu X, Hu Z, Ji H (2021). Effects of the defatted *Schizochytrium* sp. on growth performance, fatty acid composition, histomorphology and antioxidant status of juvenile mirror carp (*Cyprinus carpio var. speculari*s). J Aquac Res.

[54] Abdelghany MF, El-Sawy HB, Abd El-hameed SA, Khames MK, Abdel-Latif HM, Naiel MA (2020). Effects of dietary *Nannochloropsis oculata* on growth performance, serum biochemical parameters, immune responses, and resistance against *Aeromonas veronii* challenge in Nile tilapia (*Oreochromis niloticus*). J Fish Shellfish Immunol.

[55] Sarker PK, Kapuscinski AR, Bae AY, Donaldson E, Sitek AJ, Fitzgerald DS, Edelson OF (2018). Towards sustainable aquafeeds: Evaluating substitution of fishmeal with lipid-extracted microalgal co-product *(Nannochloropsis oculata*) in diets of juvenile Nile tilapia (*Oreochromis niloticus*). PLoS One.

[56] Mounes HAM, Mansour EG, Ahmed KM (2020). Effect of Azolla pinnata and Nannochloropsis oculata on growth performance and immunoresponse of Nile tilapia (Oreochromis niloticus) and its resistance to bacterial infection. Egypt J Aquac.

[57] Qiao H, Hu D, Ma J, Wang X, Wu H, Wang J (2019). Feeding effects of the microalga *Nannochloropsis* sp. on juvenile turbot (*Scophthalmus maximus* L.). Algal Res.

[58] Khajepour F, Hosseini SA (2012). Citric acid improves growth performance and phosphorus digestibility in Beluga (*Huso huso*) fed diets where soybean meal partly replaced fish meal. J Anim Feed Sci Technol.

[59] dos Santos SKA, Schorer M (2019). Moura GdS, Lanna EAT, Pedreira MM: Evaluation of growth and fatty acid profile of Nile tilapia (*Oreochromis niloticus*) fed with *Schizochytrium* sp. Aquac Res.

[60] Ungsethaphand T, Peerapornpisal Y, Whangchai N, Sardsud U (2010). Effect of feeding *Spirulina platensis* on growth and carcass composition of hybrid red tilapia (*Oreochromis mossambicus× O. niloticus*). J Maejo Int J Sci Technol Health Care.

[61] Li MH, Robinson EH, Tucker CS, Manning BB, Khoo L (2009). Effects of dried algae *Schizochytrium* sp., a rich source of docosahexaenoic acid, on growth, fatty acid composition, and sensory quality of channel catfish *Ictalurus punctatus*. J Aquaculture.

[62] Yang S-D, Liou C-H, Liu F-G (2002). Effects of dietary protein level on growth performance, carcass composition and ammonia excretion in juvenile silver perch (*Bidyanus bidyanus*). Aquaculture.

[63] Glencross BD (2009). Exploring the nutritional demand for essential fatty acids by aquaculture species. Rev Aquac.

[64] Zárate R, el Jaber-Vazdekis N, Tejera N, Pérez JA, Rodríguez C (2017). Significance of long chain polyunsaturated fatty acids in human health. Clin Transl Med.

[65] Brown ML, Nematipour GR, Gatlin DM (1992). Dietary protein requirement of juvenile sunshine bass at different salinities. Progressive Fish-Culturist.

[66] Habte-Tsion H-M, Kolimadu GD, Rossi W, Filer K, Kumar V (2020). Effects of *Schizochytrium* and micro-minerals on immune, antioxidant, inflammatory and lipid-metabolism status of *Micropterus salmoides* fed high-and low-fishmeal diets. Sci Rep.

[67] D’Orazio N, Gemello E, Gammone MA, De Girolamo M, Ficoneri C, Riccioni G (2012). Fucoxantin: A treasure from the sea. Mar Drugs.

[68] Kumar V, Habte‐Tsion HM, Allen KM, Bowman BA, Thompson KR, El‐Haroun E, Filer K, Tidwell JH (2018). Replacement of fish oil with *Schizochytrium* meal and its impacts on the growth and lipid metabolism of Pacific white shrimp (*Litopenaeus vannamei*). Aquac Nutr.

[69] Nasirian F, Sarir H, Moradi-Kor N (2019). Antihyperglycemic and antihyperlipidemic activities of *Nannochloropsis oculata* microalgae in Streptozotocin-induced diabetic rats. Biomol Concepts.

[70] Raji AA, Alaba PA, Yusuf H, Bakar NHA, Taufek NM, Muin H, Alias Z, Milow P, Razak SA (2018). Fishmeal replacement with *Spirulina Platensis* and *Chlorella vulgaris* in African catfish (*Clarias gariepinus*) diet: Effect on antioxidant enzyme activities and haematological parameters. Res Vet Sci.

[71] Abbas N, El-shafei R, Zahran E, Amer M (2020). Some pharmacological studies on *Chlorella vulgaris* in tilapia fish. Kafrelsheikh Vet Med J.

[72] Abdulrahman NM, Hama Ameen HJ, Hama SR, Hassan BR, Nader PJ (2019). Effect of microalgae *Spirulina* spp. as food additive on some biological and blood parameters of common carp *Cyprinus carpio* L. Iraqi J Vet Sci.

[73] Leaver MJ, Bautista JM, Björnsson BT, Jönsson E, Krey G, Tocher DR, Torstensen BE (2008). Towards fish lipid nutrigenomics: current state and prospects for fin-fish aquaculture. Rev Fish Sci.

[74] Sørensen M, Gong Y, Bjarnason F, Vasanth GK, Dahle D, Huntley M, Kiron V (2017). *Nannochloropsis oceania*-derived defatted meal as an alternative to fishmeal in Atlantic salmon feeds. PLoS One.

[75] Zahran E, Elbahnaswy S, Ibrahim I, Khaled AA (2021). *Nannochloropsis oculata* enhances immune response, transcription of stress, and cytokine genes in Nile tilapia subjected to air exposure stress. Aquac Rep.

[76] Halliwell B, Gutteridge JM (2015). Free radicals in biology and medicine.

[77] Madeira D, Narciso L, Cabral HN, Vinagre C, Diniz MS (2013). Influence of temperature in thermal and oxidative stress responses in estuarine fish. Comp Biochem Physiol A Mol Integr Physiol.

[78] Xing H, Li S, Wang X, Gao X, Xu S, Wang X (2013). Effects of atrazine and chlorpyrifos on the mRNA levels of HSP70 and HSC70 in the liver, brain, kidney and gill of common carp (*Cyprinus carpio* L.). Chemosphere.

[79] Sørensen SL, Ghirmay A, Gong Y, Dahle D, Sørensen M, Kiron V (2021). Growth, Chemical Composition, Histology and Antioxidant Genes of Atlantic Salmon (*Salmo salar*) Fed Whole or Pre-Processed *Nannochloropsis oceanica* and *Tetraselmis* sp. Fishes.

[80] Zhao W, Fang H-H, Gao B-Y, Dai C-M, Liu Z-Z, Zhang C-W, Niu J (2020). Dietary *Tribonema* sp. supplementation increased growth performance, antioxidant capacity, immunity and improved hepatic health in golden pompano (*Trachinotus ovatus*). Aquaculture.

[81] Sung YY, MacRae T. Heat shock proteins and disease control in aquatic organisms. J Aquac Res Dev S. 2011;S2-006.

[82] Tiong IKR, Nagappan T, Wahid MEA, Muhammad TST, Tatsuki T, Satyantini WH, Mahasri G, Sorgeloos P, Sung YY (2020). Antioxidant capacity of five microalgae species and their effect on heat shock protein 70 expression in the brine shrimp Artemia. Aquac Rep.

[83] Goiris K, Muylaert K, Fraeye I, Foubert I, De Brabanter J, De Cooman L (2012). Antioxidant potential of microalgae in relation to their phenolic and carotenoid content. J Appl Phycol.

[84] Custódio L, Justo T, Silvestre L, Barradas A, Duarte CV, Pereira H, Barreira L, Rauter AP, Alberício F, Varela J (2012). Microalgae of different phyla display antioxidant, metal chelating and acetylcholinesterase inhibitory activities. Food Chem.

[85] Bauer G, Zarkovic N (2015). Revealing mechanisms of selective, concentration-dependent potentials of 4-hydroxy-2-nonenal to induce apoptosis in cancer cells through inactivation of membrane-associated catalase. Free Rad Biol Med.

[86] Wu K, Cleveland BM, Portman M, Sealey WM, Lei XG (2020). Supplemental microalgal DHA and astaxanthin affect astaxanthin metabolism and redox status of Juvenile Rainbow trout. Antioxidants.

[87] Zhu S, Portman M, Cleveland BM, Magnuson AD, Wu K, Sealey W, Lei XG (2021). Replacing fish oil and astaxanthin by microalgal sources produced different metabolic responses in juvenile rainbow trout fed 2 types of practical diets. J Anim Sci.

[88] Dash P, Patel S, Dixit A, Garg LC, Sahoo PK (2015). Four pro-inflammatory cytokines of rohu (*Labeo rohita)* during early developmental stages, their tissue distribution and expression by leucocytes upon in-vitro stimulation. Fish Shellfish Immunol.

[89] Mahmoud MM, El-Lamie MM, Kilany OE, Dessouki AA (2018). *Spirulina* (*Arthrospira platensis*) supplementation improves growth performance, feed utilization, immune response, and relieves oxidative stress in Nile tilapia (*Oreochromis niloticus*) challenged with *Pseudomonas fluorescens*. Fish Shellfish Immunol.

[90] Patterson E, Wall R, Fitzgerald G, Ross R, Stanton C. Health implications of high dietary omega-6 polyunsaturated fatty acids. J Nutr Metab. 2012. 16 pages. Aricle ID 539426. 10.1155/2012/539426 PMC3335257 22570770

[91] Nayak S, Khozin-Goldberg I, Cohen G, Zilberg D (2018). Dietary supplementation with ω6 LC-PUFA-rich algae modulates zebrafish immune function and improves resistance to streptococcal infection. Front Immunol.

[92] Aste-Amezaga M, Ma X, Sartori A, Trinchieri G (1998). Molecular mechanisms of the induction of IL-12 and its inhibition by IL-10. J Immunol.

[93] Zahran E, Awadin W, Risha E, Khaled AA, Wang T (2019). Dietary supplementation of *Chlorella vulgaris* ameliorates chronic sodium arsenite toxicity in Nile tilapia *Oreochromis niloticus* as revealed by histopathological, biochemical and immune gene expression analysis. Fish Sci.

[94] Ayisi CL, Zhao J-L (2017). Fatty acid composition, lipogenic enzyme activities and mRNA expression of genes involved in the lipid metabolism of Nile tilapia fed with palm oil. Turkish J Fish Aquat Sci.

[95] Dong G-F, Zou Q, Wang H, Huang F, Liu X-C, Chen L, Yang C-Y, Yang Y-o (2014). Conjugated linoleic acid differentially modulates growth, tissue lipid deposition, and gene expression involved in the lipid metabolism of grass carp. Aquaculture.

[96] Ntambi JM (1999). Regulation of stearoyl-CoA desaturase by polyunsaturated fatty acids and cholesterol. J Lipid Res.

[97] Kersten S (2014). Integrated physiology and systems biology of PPARα. Mol Metab.

[98] Luo L, Wei H, Ai L, Liang X, Wu X, Xing W, Chen P, Xue M (2019). Effects of early long-chain n-3HUFA programming on growth, antioxidant response and lipid metabolism of Siberian sturgeon (*Acipenser baerii Brandt*). Aquaculture.

[99] Zhao W, Fang HH, Liu ZZ, Chen JM, Zhang CW, Gao BY, Niu J (2021). Responses in growth performance, enzymatic activity, immune function and liver health after dietary supplementation of *Porphyridium* sp. in juvenile golden pompano (*Trachinotus ovatus*). Aquac Nutr.

[100] Zuo R, Mai K, Xu W, Turchini GM, Ai Q (2015). Dietary ALA, But not LNA, Increase Growth, Reduce Inflammatory Processes, and Increase Anti‐Oxidant Capacity in the Marine Finfish Larimichthys crocea: Dietary ALA, but not LNA, Increase Growth, Reduce Inflammatory Processes, and Increase Anti‐oxidant Capacity in the Large Yellow Croaker. Lipids.

[101] Michalik L, Auwerx J, Berger JP, Chatterjee VK, Glass CK, Gonzalez FJ, Grimaldi PA, Kadowaki T, Lazar MA, O'Rahilly S (2006). International Union of Pharmacology. LXI. Peroxisome proliferator-activated receptors. Pharmacol Rev.

[102] Tocher DR (2003). Metabolism and functions of lipids and fatty acids in teleost fish. Rev Fish Sci.

[103] Olsvik P, Torstensen B, Berntssen M (2007). Effects of complete replacement of fish oil with plant oil on gastrointestinal cell death, proliferation and transcription of eight genes’ encoding proteins responding to cellular stress in Atlantic *salmon Salmo salar* L. J Fish Biol.

[104] Dezfuli BS, Giari L, Lui A, Squerzanti S, Castaldelli G, Shinn AP, Manera M, Lorenzoni M (2012). Proliferative cell nuclear antigen (PCNA) expression in the intestine of *Salmo trutta trutta* naturally infected with an acanthocephalan. Parasit Vectors.

[105] Ortego LS, Hawkins WE, Walker WW, Krol RM, Benson WH (1995). Immunohistochemical detection of proliferating cell nuclear antigen (PCNA) in tissues of aquatic animals utilized in toxicity bioassays. Marine Environ Res.

[106] Leung AY, Leung JC, Chan LY, Ma ES, Kwan TT, Lai K, Meng A, Liang R (2005). Proliferating cell nuclear antigen (PCNA) as a proliferative marker during embryonic and adult zebrafish hematopoiesis. Histochem Cell Biol.

[107] Reis B, Gonçalves AT, Santos P, Sardinha M, Conceição LE, Serradeiro R, Pérez-Sánchez J, Calduch-Giner J, Schmid-Staiger U, Frick K (2021). Immune Status and Hepatic Antioxidant Capacity of Gilthead Seabream *Sparus aurata* Juveniles Fed Yeast and Microalga Derived β-glucans. Mar Drugs.

[108] Borowitzka MA (1997). Microalgae for aquaculture: opportunities and constraints. J Appl Phycol.

[109] Haas S, Bauer J, Adakli A, Meyer S, Lippemeier S, Schwarz K, Schulz C (2016). Marine microalgae *Pavlova viridis* and *Nannochloropsis* sp. as n-3 PUFA source in diets for juvenile European sea bass (*Dicentrarchus labrax* L.). J Appl Phycol.

[110] Eynon B (2016). Assessment of novel algal biomass sources as potential ingredients in diets for tilapia (*Oreochromis niloticus*).

